# Molecular characterisation of atypical BSE prions by mass spectrometry and changes following transmission to sheep and transgenic mouse models

**DOI:** 10.1371/journal.pone.0206505

**Published:** 2018-11-08

**Authors:** Adriana Gielbert, Jemma K. Thorne, Jane M. Plater, Leigh Thorne, Peter C. Griffiths, Marion M. Simmons, Claire A. Cassar

**Affiliations:** Animal and Plant Health Agency-Weybridge, Addlestone, Surrey, United Kingdom; INRA Centre de Jouy-en-Josas, FRANCE

## Abstract

The prion hypothesis proposes a causal relationship between the misfolded prion protein (PrP^Sc^) molecular entity and the disease transmissible spongiform encephalopathy (TSE). Variations in the conformation of PrP^Sc^ are associated with different forms of TSE and different risks to animal and human health. Since the discovery of atypical forms of bovine spongiform encephalopathy (BSE) in 2003, scientists have progressed the molecular characterisation of the associated PrP^Sc^ in order to better understand these risks, both in cattle as the natural host and following experimental transmission to other species. Here we report the development of a mass spectrometry based assay for molecular characterisation of bovine proteinase K (PK) treated PrP^Sc^ (PrP^res^) by quantitative identification of its N-terminal amino acid profiles (N-TAAPs) and tryptic peptides. We have applied the assay to classical, H-type and L-type BSE prions purified from cattle, transgenic (Tg) mice expressing the bovine (Tg110 and Tg1896) or ovine (TgEM16) prion protein gene, and sheep brain. We determined that, for classical BSE in cattle, the G96 N-terminal cleavage site dominated, while the range of cleavage sites was wider following transmission to Tg mice and sheep. For L-BSE in cattle and Tg bovinised mice, a C-terminal shift was identified in the N-TAAP distribution compared to classical BSE, consistent with observations by Western blot (WB). For L-BSE transmitted to sheep, both N-TAAP and tryptic peptide profiles were found to be changed compared to cattle, but less so following transmission to Tg ovinised mice. Relative abundances of aglycosyl peptides were found to be significantly different between the atypical BSE forms in cattle as well as in other hosts. The enhanced resolution provided by molecular analysis of PrP^res^ using mass spectrometry has improved insight into the molecular changes following transmission of atypical BSE to other species.

## Introduction

Classical bovine spongiform encephalopathy (C-BSE) is a prion disease of cattle, first described in 1986 [1, 2], and identified by subsequent epidemiological studies as an extended common source epidemic linked to meat and bone meal in cattle feed following changes to rendering practices [3]. An EU-wide total ban on the use of meat and bone meal as a feed supplement followed, and specified risk material was removed from slaughtered cattle (Regulation EC No. 999/2001). Additionally, systematic active surveillance for transmissible spongiform encephalopathy (TSE) in cattle and small ruminants was introduced across the EU. This intensive scrutiny led to atypical forms of BSE being identified in cattle. These atypical forms are commonly referred to as H-BSE and L-BSE based on the higher or lower molecular mass of their nongycosylated fragments in Western blots [4]. First reported in France (H-BSE) [5] and Italy (L-BSE) [6], atypical BSE was identified in other countries in Europe as well as in Japan, the US, Canada and Brazil [7, 8]. Atypical BSE occurs sporadically and it has been hypothesised that cases may arise spontaneously, as may have been the case initially for C-BSE. To date, C-BSE is the only prion disease of animal origin confirmed to be zoonotic, giving rise to variant Creutzfeldt Jakob Disease in humans [9–11]. However, under experimental circumstances, both atypical BSE types (H-BSE and L-BSE) are transmissible to a range of species, therefore a hypothetical risk to the food and feed chains remains. Transmission experiments to (transgenic) mice [12–16], sheep [17, 18] and primates [19–21] have been carried out to improve understanding of species barriers and to determine phenotypes. Mouse transmission studies have shown that both H- and L-BSE can acquire properties indistinguishable from C-BSE [15, 16, 22]. L-BSE is more rapidly transmissible to primates than C-BSE [19, 21] and to humanised mouse models with less of a transmission barrier than C-BSE [14], suggestive of a higher zoonotic potential for L-BSE compared to C-BSE. Experimental transmission of L-BSE to sheep has been achieved and, unlike C-BSE in sheep, it presents as a novel TSE phenotype in sheep with regard to both neuropathology and clinical signs [18]. While the zoonotic potential of this novel TSE phenotype has not been established, the precautionary principle entails that the current measures to protect the food chain from TSEs need to be maintained indefinitely [23].

The misfolded prion protein, PrP^Sc^, is considered causative of prion disease and remains its most reliable diagnostic marker. The prion hypothesis connects the molecular entity of the misfolded PrP protein with the strain, changes in PrP^Sc^ conformation point to changes in biological properties of a TSE, and vice versa [24–27]. Different forms of TSE are associated with different conformers of PrP^Sc^. Characterisation of atypical BSE by Western blot (WB) analysis of the associated PrP^Sc^ has provided a classification into two overall types, which are described in relation to C-BSE. WB analysis of C-BSE produces a characteristic three-band pattern of proteinase-K treated PrP^Sc^ (PrP^res^), where the band with the lowest molecular mass (MM), approximately 19 kDa, corresponds to the nonglycosylated isoform, the second band to the two monoglycosylated isoforms and the third band to the diglycosylated isoform. The nonglycosylated PrP^res^ band for L-BSE is found to migrate to a slightly lower MM than for C-BSE and, like C-BSE, lacks reactivity with “Group A” [28, 29] monoclonal antibodies such as 12B2 and P4, the epitopes of which are just N-terminal to the WNK sequence (residues 102–105 for 5 octarepeats as analogous to ovine PrP) [4]. The nonglycosylated PrP^res^ band for H-BSE is found to migrate to a slightly higher MM than for C-BSE, and does show reactivity with “Group A” antibodies. Additionally, atypical BSEs can be biochemically distinguished from C-BSE by differences in the relative abundance of the three glycoforms bands, where the relative abundance of the diglycosylated band is higher for C-BSE compared to either H-BSE [5] or L-BSE [6]. Finally, H-BSE reveals an additional 3-band pattern at lower MM, where the nonglycosylated runs at approx. 14 kDa, when detected with a “Group C” monoclonal antibody such as SAF84 (epitope RPVDQY) or 94B4 (epitope HTVTTTTK) [28, 29].

The enhanced resolution provided by mass spectrometry (MS) has the potential to provide an even better insight into prion conformational differences than already achieved with WB. MS has been previously used to detect differences between prions from different strains [30–32] and at APHA we have developed an assay using the MS based detection technique “multiple Selected Reaction Monitoring” (mSRM) to study the diversity of ovine prions [33–35]. Application of mSRM to PrP^res^ marker peptides prepared from brain samples allows the detection and quantification of different N-terminal cleavage sites. Together these provide a high resolution N-terminal amino acid profile (N-TAAP) of the “ragged” N-terminus of PrP^res^ from a given sample. Comparing the N-TAAPs of samples from different TSEs can in principle reveal more subtle differences than can be resolved by WB. Similar analysis of fully tryptic peptides from the protease resistant part of PrP^Sc^ can enhance insight into its structural properties. Here we report the development of such a bovine PrP^res^ peptide assay as well as its application to identify differences between the N-TAAPs as well as tryptic peptide profiles of C-, H- and L-BSE prions from cattle and from transgenic mouse lines expressing the bovine (Tg bov) and or ovine (Tg ov) prion protein gene, and of C- and L-BSE from sheep.

## Methods

### Samples

Frozen brain tissue samples from cattle, sheep and mice naturally or experimentally infected with TSEs were obtained from the Animal and Plant Health Agency (AHPA) archives. A diagnosis of TSE was confirmed by detection of vacuolation and detection of abnormal prion protein by immunohistochemistry and Western blotting. Brain samples were collected post-mortem and stored at –80°C prior to use for mass spectrometry.

An overview of the provenance of samples used in this study is given in Table 1. Brain stem samples from natural C-BSE (n = 10) were obtained from United Kingdom (UK) field surveillance activities. Brain stem samples from experimental C-BSE cases (n = 4) were primary passages following oral administration of inoculum SE1736 (BBP1) to cattle [36]. Brain stem from four experimental H-type (designated H1-4) and 2 L-type (L2 and L4) bovine BSE cases originated from primary passage by intracerebral challenge, as described previously [37]. Two further experimental L-type BSE samples (L5 and L6) resulted from secondary passage, also described previously [38]. Four samples of ovine L-BSE brain stem were obtained from four animals challenged with the same inoculum used to produce bovine L-BSE described above [18]. The ovine C-BSE sample was obtained by oral transmission of 5g cattle BSE homogenate [39] and the ovine CH1641 sample by intracerebral inoculation of the sequentially passaged CH1641 isolate [33].

**Table 1:** Provenance of samples included in this study.

Sample ref.	TSE	Host*	Transmission route (amount)	Inoculum	Figure	Reference
2125/03	C-BSE	Cattle	Natural	n/a	-	
0401/04						
1093/02						
914/07						
0864/04						
02/00921						
0351/04						
06/06990					Fig 2 & S3 Fig	[36]
1643/97						
1339/96						
022/02			OD (100g)	SE1736 (BBP1)	-	
026/02			OD (100g)			
969/04			OD (1g)			
298/05			OD (1g)			
H1	H-BSE		IC (1ml 10%)	French H-type ESB-H-07-0644	Fig 3 & S4 Fig	[37]
H2						
H3						
H4						
L2	L-BSE			BP12 (Italian L-type case 141387/02)	Fig 3 & S4 Fig	[37]
L4						
L5				BP24 (L1)	Fig 3 & S4 Fig	[38]
L6				BP25 (L4)		
456/11		ALRQ/VRQ		BP12 (Italian L-type case 141387/02)	Fig 7 & S8 Fig	[18]
140/11		ALRQ/VRQ				
267/11		VRQ/VRQ				
457/11		AFRQ/AFRQ				
M1	C-BSE	Tg110	IC (20μl 10%)	SE1867/18 (UK C-BSE 05/00094)	Fig 4 & S5 Fig	
M2						
M3		Tg1896				
M4						
M13		TgEM16			S9 Fig	
M14						
M5	H-BSE	Tg110		SE2014/63 (UK H-BSE 09/00015)	Fig 5 & S6 Fig	[40]**
M6						
M7		Tg1896				
M15		TgEM16			S9 Fig	
M16						
M9	L-BSE	Tg110		SE2014/62 (UK L-BSE 11/00008)	Fig 6 & S7 Fig	[41]**
M10						
M11		Tg1896				
M12						
M17		TgEM16			S9 Fig	
M18						
822/09		AHQ/AHQ	OD	BBP1	S9 Fig	[35, 39]
851/05	CH1641	AHQ/AHQ	IC	CH1641-VLA1	S9 Fig	[33, 35]

* Ovine host if only genotype (136 A/V, 154R/H, (141L/F), 171Q/H) is given. 141L/F is specified only in the context of the 136A 154R 171Q allele, to which this variant is exclusive.

** These publications relate to the inoculum source only

Whole mouse brain homogenates were prepared following the transmissions of C-, H- and L-BSE to two lines of Tg bov mice, Tg110 [42] and Tg1896 (Defra project SE1753, [43]), and one line of Tg ov mice, TgEM16 (A^136^H^154^Q^171^) [44], as part of larger bioassay studies (Defra projects SE1867 and SE2014). Western blot analysis to confirm diagnosis was carried out using Sha31 and SAF84 antibodies (S1 Fig). In most cases 200–300 mg tissue fractions were used for the preparation of duplicate tryptic digests for analysis by mass spectrometry, except for L-BSE in Tg110, where approx. 100 mg was used.

### Animal studies

Cattle and sheep were inoculated intracerebrally or orally in the context of previous studies, as described elsewhere [18, 33, 36–38]. Mice were inoculated intracerebrally with 20 μl of TSE inoculum prepared from single-brain material at 10% (w/v) in saline as described previously [45]. All animal procedures were conducted in accordance with the UK Animal (Scientific Procedures) Act 1986 under license from the Government Home Office (Project Licence numbers 70/6781, and 70/7167 and 70/6892, for ruminant and murine procedures, respectively). Such licences are only granted following approval by the internal Animal and Plant Health Agency (APHA) ethical review process as mandated by the Home Office.

### Sample processing for analysis by mass spectrometry

All samples were prepared and processed in our laboratories at UK Advisory Committee for Dangerous Pathogens Containment Level 3 with derogation for TSEs.

All chemicals were of reagent grade or better and obtained from Sigma-Aldrich unless otherwise stated. Brain tissues were weighed and individually ground in a sample homogenizer (Bio-Rad) at 6.5 Hz for a 45 s cycle, to a 20% homogenate in 10% N-lauroyl sarcosinate in 0.01 M PBS pH 7.4 (BLB) containing protease inhibitor (cOmplete ULTRA tablets, Mini, EDTA free, Roche Diagnostics). Subsequently, homogenates were diluted to 10% by addition of further BLB. For the study where data from 350 and 1000 mg C-BSE tissue quantities were compared, 10% homogenates were prepared directly using an Omni homogenizer. The 10% homogenates were first centrifuged at 17,000 g for 2 min; subsequently the supernatant was selected and centrifuged at 338,000 g for 30 min. The resulting pellets were suspended in water (200 μl) to which 15% KI in 0.01 M Tris-HCl (pH 7.4) containing 1.5% sodium thiosulfate and 1% N-lauroyl sarcosinate (400 μl) was added. At this point, suspensions were divided into 2x300 μl aliquots which were processed and analyzed in parallel. To each 300 μl aliquot, 12 μl Proteinase K solution (39 units/mg, 1 mg/ml in water) was added. Following incubation at 37°C for 30 min under agitation (Thermoshaker, 1000 rpm), 12 μl Pefablock solution (3 mg/ml) was added to stop the reaction. This solution containing PrP^res^ was processed further by addition of 325 μl of a 1-propanol/1-butanol (1/1) mixture, following which suspensions were vortex-mixed and centrifuged at 24,000 g for 30 min. Pellets were suspended in water (100 μl), 1 M NaCl was added (900 μl), then suspensions were vortex-mixed and centrifuged for 10 min at 15,000 g. Where parallel analysis by Western blotting was carried out, pellets were suspended in water (40 μl), an aliquot was taken for Western blot analysis (20 μl), and more water (80 μl) was added to the remaining suspensions. To all 100 μl suspensions, 1 M NaCl was added (900 μl), then suspensions were vortex-mixed and centrifuged for 10 min at 15,000 g.

The resulting pellets were solubilized in guanidine hydrochloride (GuHCl, 100 μl, 6M in 50 mM Tris, pH 8.0), PrP^res^ in the pellets was reduced with 2mM 1,4-dithioerythritol at 95°C for 20 min and alkylated with 4-vinylpyridine (10% in water, 10 μl) for 10 min at ambient temperature. Insoluble material was discarded following centrifugation (2 min, 11,000 g) and protein was isolated from the supernatant following precipitation with cold methanol (900 μl, -20°C) maintaining -20°C overnight before centrifugation at 10,000 g for 10 min at -4°C. The supernatant was discarded and the pellet resuspended in cold methanol (100 μl, -20°C), centrifuged at 10,000 g for 2 min at -4°C and after discarding of the supernatant the pellet was allowed to dry at ambient temperature for 20 min. The pellet was suspended in 10 μl freshly prepared urea (9M). Subsequently 10 μl of a buffer consisting of 150 mM Trizma base, 60 mM methylamine-HCl and 15 mM calcium acetated adjusted to pH 8.3 with glacial acetic acid, and 2 μl of the synthetic trypsin substrate boc-vla-leu-lys-7-amido-4-methylcoumarin (1 ng/μl) were added. Trypsin (sequencing grade, Promega) was dissolved in buffer in accordance with the manufacturer’s instructions (100 ng/μl,) and 3μl was added to each PrP^res^ preparation. Following trypsin digestion at 30°C for 18 h, the reaction was terminated by addition of 12 μl 5% v/v formic acid (FA). Thus, each 300 mg sample of brain gave rise to two replicates of PK-treated and trypsin digested PrP^res^ preparations. Digests that could not be analyzed by chip-HPLC mSRM within 18 h of this final preparation step (see Results) were stored at -20°C until analysis.

### Chip-HPLC-MS analysis

**Fig 1:**
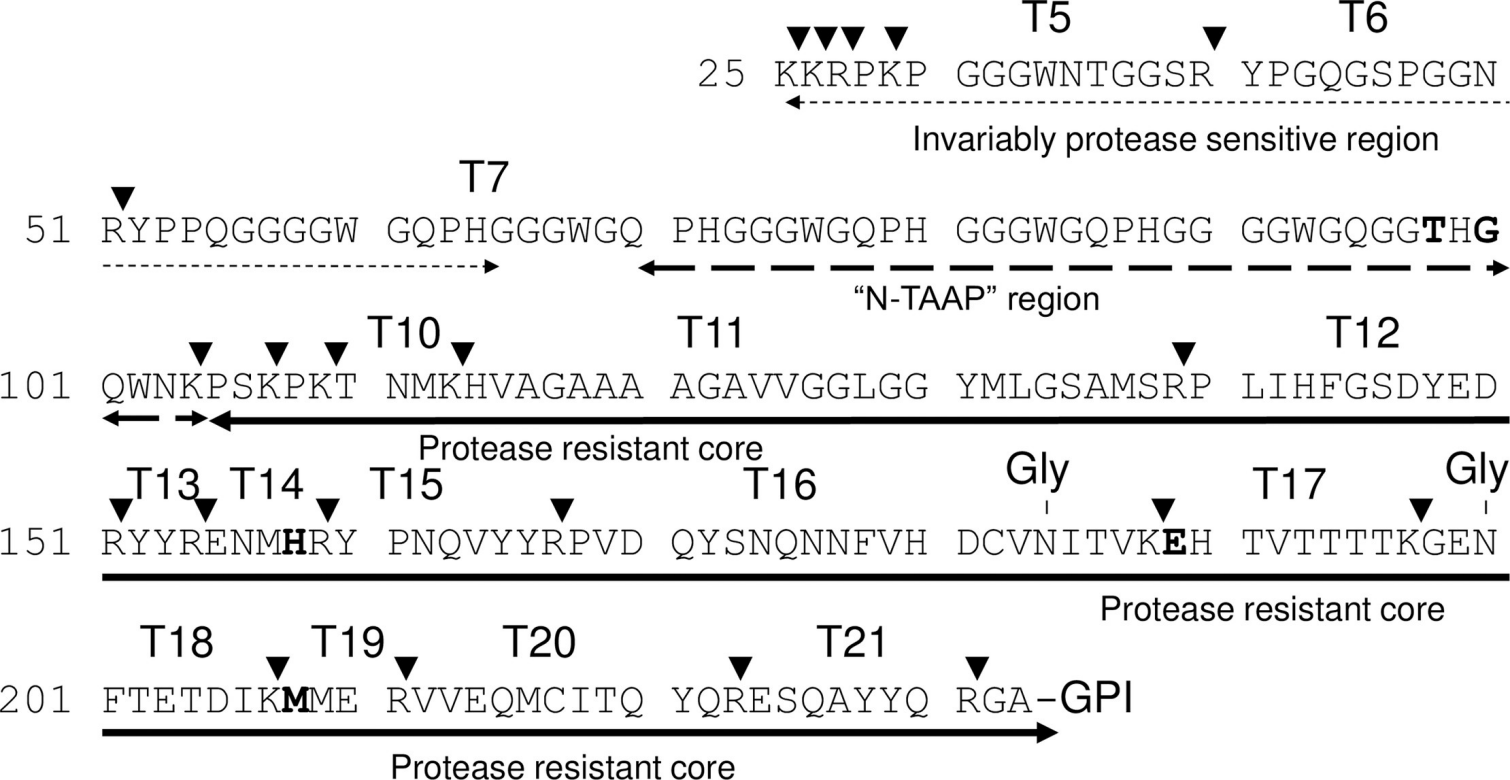
Bovine PrP protein sequence. N-TAAP region and tryptic peptides used in the mass spectrometry-based assay are indicated (also refer to S1 and S4 Tables). A five-octarepeat version is shown to facilitate analogy with ovine sequence. Arrowheads indicate trypsin cleavage sites. Amino acid residues in **bold** indicate interspecies polymorphisms in comparison with the ovine sequence.

Since there are sequence differences between ovine and bovine prions that result in mass differences between a large number of PrP^Sc^ marker peptides (Fig 1, S2 Fig and S1 and S2 Tables), a different assay is required to carry out similar studies with bovine prions than previously published for ovine prions [33–35]. Analogues of peptides representing relevant sequences of bovine PrP (Fig 1 and S1 and S2 Tables) including those representing post-translational modifications pyroglutamylation or deglycosylation, were custom synthesized (min. 98% purity; Peptide Protein Research Ltd., Eastleigh, UK) and used, without further purification, for method optimization and as external calibration standards for quantification. A multiplexed LC-MS/MS method was developed using an Agilent 6410 triple quadrupole mass spectrometer interfaced with a Chip Cube and Agilent 1200 nano-HPLC system (Agilent, UK). A “dynamic MRM” method was developed based on synthetic versions of each of the peptides to be included in the bovine PrP assay; MS/MS data were acquired for each peptide scanning an appropriate mass range, and two to four precursor/product ion pairs (transitions) selected and optimized (S3 Table). The previously developed assay for ovine PrP [35] was used to analyse ovine and ovine transgenic mouse samples (see S2 Fig for the equivalent ovine protein sequence and tryptic peptide numbering; ovine peptides used in the assay are also given S1 and S2 Tables). For brevity, the N-TAAP peptides are referred to solely by their N-terminal amino acid residue in the following, rather than by the N-terminal and C-terminal residue (for example, G77 rather than G77-K109).

Peptide calibration standards or tryptic digest samples were injected (1 μl) onto an Agilent Ultra-high Capacity chip, containing a 500 nl enrichment column and a 150mmx75μm analytical column packed with Zorbax 80-SB-C18, particle size 5 μm. For the loading pump 0.1% FA in water (LC-MS grade, Fisher Scientific) was used (3 μl/min). The chip injection flush volume was set to 8 μl. Analyte separation and elution into the mass spectrometer was carried out in forward flush mode. A gradient was used running from 3% to 50% solvent B (where solvent A was 0.1% v/v FA in water and solvent B was 0.1% FA in 90% acetonitrile and 10% water (LC-MS grade, Fisher Scientific) over 25 min at a flow rate of 400 nl/min. The capillary voltage was set to 1900 V.

Retention times were determined using the PrP peptide standards and retention time windows set at 2 min for most peptides; for peptides which tended to have wider peaks or peaks that tended to shift as the assay progressed, up to 4 min were used.

Dilution series of calibration and quality control (QC) standards ranging between 0.1 fmol/μl to 0.5 pmol/μl were run at the start of the sample batch; additional QC standards at the end and, depending on the number of preparations analyzed, mid-batch. To minimize carryover effects, duplicate injections of 8 μl 70/30 (v/v) acetonitrile/water followed by two blank runs were inserted after the highest concentration standards and between preparations from different samples. All cattle samples reported here have been analysed as part of a single HPLC-MS analysis sequence while the Tg bovinised mouse samples were analysed together as part of a different sequence, but using the same HPLC column and set of calibration standards. The ovine L-BSE samples were analysed together in a single HPLC-MS analysis sequence specific for ovine PrP, while Tg ovinised mouse preparations were analysed in a separate sequence which did not include calibration standards. The ovine C-BSE and CH1641 data included for comparison were from previous analyses [35].

### Data processing and presentation

LC-mSRM data were acquired and analyte concentrations calculated based on peak area, by interpolation from calibration curves generated, using proprietary software (Agilent MassHunter Quantitative Analysis version B.05.02). All analyte and calibration standard peaks were manually verified and re-integrated as necessary. Linear regression with none or a 1/x weighting was used, whichever gave the better fit for a given peptide (R2 > 0.98). Lower limits of detection and quantification (LoDs and LoQs) and imprecision of quantification were established using synthetic peptide mixtures of known concentration (quality controls). The LoD was defined as the concentration above which the signal-to-noise ratio exceeded 3. The LoQ was defined for each peptide as the concentration above which the calculated value was within 20% of the theoretical value (n = 2–4 depending on batch size). Values for the bovine assay are given in S3 Table; for the ovine assay as previously published [35]. Calculated peptide concentrations were transferred to GraphPad Prism v.7 for the production of N-TAAP plots. Values from processing replicates were combined to display a mean with standard deviation. Peptide relative abundances and ratios were calculated using Microsoft Excel 2010 and data transferred to GraphPad Prism v.7 for further processing and presenting. P-values were calculated by applying an unpaired two-tailed t-test as available in the Graphpad software.

The maximum tissue equivalent (TE_max_) values, given in the figure captions, represent the maximum amount of tissue from which the corresponding PrP^res^ profile was derived, supposing no losses occurred during preparation. The values were calculated on the basis that for each analysis, 1 μl was injected on column from a total of 25 μl tryptic digest, and that this digest originated from a known amount of brain tissue divided in two or four (C-BSE samples) processing replicates following homogenisation.

## Results

### Bovine C-BSE PrP^res^ profiles are uniform with predominant PK cleavage at G96

**Fig 2:**
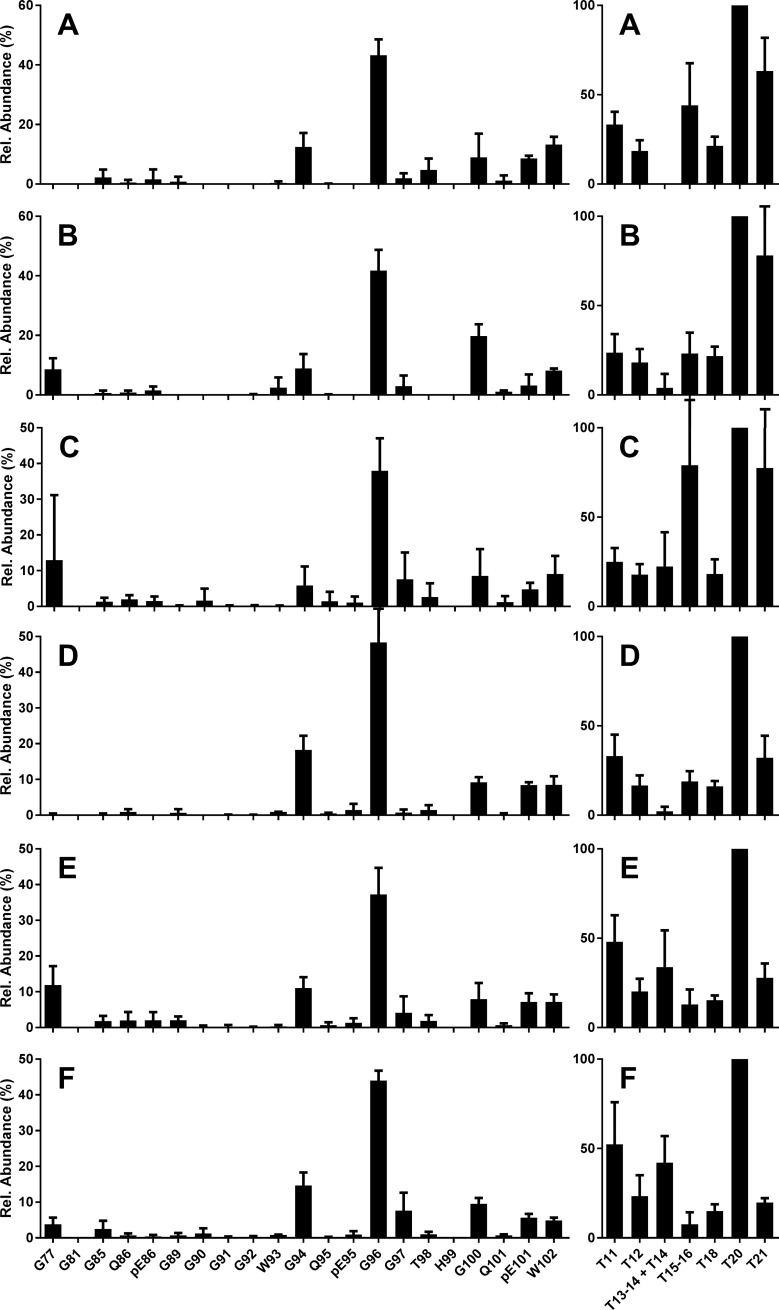
Bovine classical BSE, natural cases from the UK. N-TAAP (left-hand panels) and tryptic peptide profiles (right hand panels). Case references (A, D) 06/06990, (B, E) 1643/97, (C, F) 1339/96. Samples (A-C) were prepared using 350 mg, (D-F) using 1000 mg starting material, divided in four prior to PK treatment, and processed and analysed in parallel, giving TE_max_ of 3.5 and 10 mg, resp., then data were combined to create the profiles. Where error bars exceeded the maximum of the y-axis range displayed, they were clipped by the software and drawn in manually. Peptide numbering as in Fig 1.

When we first applied the MS-based assay to quantify bovine PrP^res^ peptides prepared from C-BSE infected brain stem, we found that the absolute peptide abundances obtained from bovine tissue were much lower compared to equivalent amounts of ovine tissue [35]. To assess whether the resulting peptide profiles were nonetheless of sufficient quality, we compared results obtainable from the smaller amounts of starting material with those based on larger quantities. A total of 24 tryptic digests were prepared by dividing homogenates, each prepared from 350 mg or 1000 mg brain stem from three different C-BSE field cases, into four aliquots. Each homogenate aliquot gave rise to a final volume of 25 μl tryptic digest. Hence each chip-HPLC-MS analysis (1 μl digest injected on column) corresponded to a maximum tissue equivalent (TE_max_) of 3.5 mg or 10 mg. Yields of tryptic peptide T20 (VVEQMCITQYR or V212-R223, see Fig 1 and S2 Table for tryptic peptide numbering), used as a marker for [PrP^res^], were 2–3 fmol/μl for 350 mg brain stem (Figures A-C in S3 Fig) and 10–15 fmol/μl for 1000 mg brain stem (Figures D-F in S3 Fig). This equates to an approximate linear correlation between yield and amount of starting material. While the error bars in the absolute profiles (S3 Fig) appeared larger for 350 mg compared to 1000 mg brain stem used, error bars were reduced once relative abundances were calculated giving rise to the final N-TAAP and tryptic profiles, and are similar for 350 mg and 1000 mg starting material (Fig 2).

The N-TAAPs for C-BSE were consistent between the different field case samples and amounts (Fig 2 and S3 Fig). The fragment with N-terminal G96 was the most abundant (approx. 40% of the total), followed by G94 (10–20%), and then G100, the pyroglutamyl form of residue 101 (pE101), and W102 (each approx. 10%). The presence of pE101 without similar abundance of Q101 fragments suggested that some of the PrP^Sc^ fragments existed prior to PK treatment. These observations are consistent with WB results where C-BSE is known to lack reactivity with Group A monoclonal antibodies (mAbs) such as P4 and 12B2 which have epitopes N-terminal to G94. The other bovine C-BSE preparations analysed in this study, from surveillance cases as well as experimental transmissions (Table 1), gave results similar to those in Fig 2 and S3 Fig.

T20 was consistently the most abundant of the tryptic peptides (S3 Fig, right-hand panels). To obtain more consistent tryptic peptide profiles when averaged over multiple preparations, abundances were calculated relative to T20 in individual preparations and averaged for the replicates (Fig 2, right-hand panels). The levels of the aglycosyl forms of T15-16 (Y160-R188) and T18 (G198-K207) were similar at approx. 20% of the abundance of T20 (Fig 2). Note that fully tryptic peptides T15 (Y160-R167) and T16 (P168-R188) are only observed at very low abundance (<0.01% of T15-16). Cleavage of this R-P bond does not appear to be favoured by trypsin, in contrast to the R-P bond between T11 (H114-R139) and T12 (P140-R151) [35]. Therefore T15 and T16 have been disregarded in the remainder of this work.

The relative abundance of C-terminal peptide T21 (E224-R231) was reduced in the profiles based on the larger amounts of starting material (compare right hand panels in Figures A-C in S3 Fig with those in Figures D-F in S3 Fig). This was attributed to chromatographic displacement: the low hydrophobicity of T21 (calculated MEEK index at pH2.1 is 2.2 [46]) leads to a reduced interaction with the column stationary phase for higher total peptide concentrations, causing partial elution into the column void.

We concluded that consistent N-TAAP and tryptic peptide profiles could in principle be obtained from bovine BSE tissue quantities of approx. 350 mg, but decided to divide these into two (TE_max_ = 7 mg) rather than four processing aliquots to increase the probability that the absolute PrP^res^ yield exceeded 2 fmol/μl. The use of larger amounts of starting material was considered undesirable as this might distort the profiles due to chromatographic displacement.

### Bovine atypical BSEs produce distinctive PrP^res^ peptide profiles

A total of four H-BSE (H1-4) and four L-BSE samples (L2, L4, L5 and L6) were prepared and analysed (TE_max_ = 7 mg) [37, 38]. Samples H1 and H2 contained insufficient PrP to allow N-TAAPs to be determined, despite the concentrations of tryptic peptide T20 being approx. 4 fmol/μl for H1 and 2 fmol/μl for H2. Samples H3 yielded approx. 20 fmol/μl T20 and H4 8 fmol/μl (S4 Fig) and contained sufficient N-TAAP peptides to allow profiles to be determined. Thus the order of PrP^res^ yield (highest to lowest) here is H3, H4, H2, and H1, the latter being the least abundant. This is somewhat different from the WB results reported previously where H2 was the most abundant followed by H1, H3 and H4 [37]. PrP^res^ yields for L-BSE also varied between approx. 10 and 30 fmol/μl based on T20 abundance. The order of abundance by MS was L4, L3, L6 and L5, roughly agreeing with the order reported by WB as L4, L3, L2, L1 [37] and L5, L6 [38].

**Fig 3:**
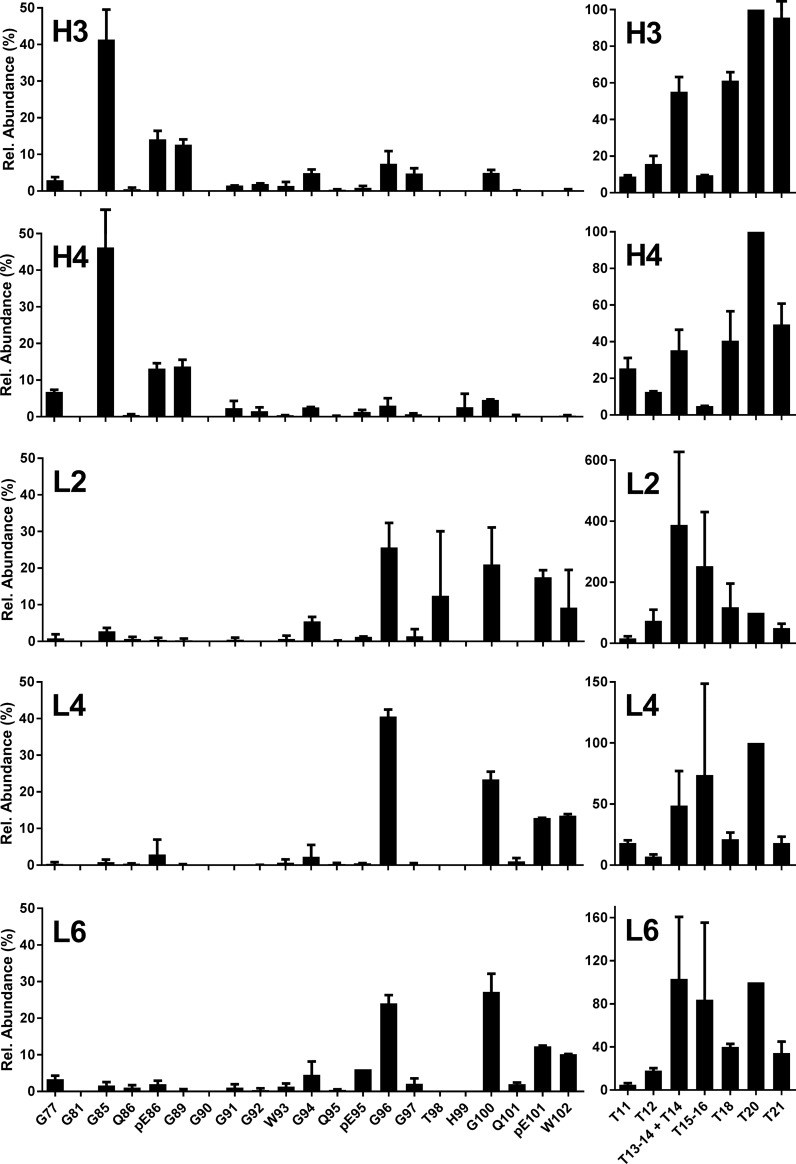
Bovine experimental atypical BSE. N-TAAP (left-hand panels) and tryptic peptide profiles (right hand panels). H3, H4: H-BSE primary passage cases [37]; L2, L4: L-BSE primary passage cases [37]; L6: L-BSE secondary passage case [38]. Samples (350 mg) were divided into two replicates prior to PK treatment and processed and analysed in parallel (TE_max_ = 7 mg), then data combined to create the profiles. Where error bars exceeded the maximum of the y-axis range displayed, they were clipped by the software and drawn in manually.

The dominant PK cleavage site for H-BSE was N-terminal to G85 (40–50%), while some pE86 and G89 N-terminal fragments were also observed (approx. 15% each) as well as G77 (5%) (Fig 3 H3 and H4). Further C-terminal cleavages were also detected, mainly G94, G96 and G100 at approx. 5% abundance, as well as G90, G91 and G92 but at even lower levels. For the L-type BSE samples (Fig 3 L3, L4 and L6), the N-terminal fragment G96 was on the whole the most abundant (20–40%), closely followed by G100 (20–30%), pE101 (15–20%) and W102 (10%). This differs from the profiles observed for C-BSE, where further C-terminal fragments were minor compared to G96. Furthermore, the abundance of G94 was much lower for L-BSE compared to C-BSE. This is the first time that the precise differences in composition of the “ragged” N-terminus of PrP^res^ between L-BSE and C-BSE have been reported. These observations are consistent with previous WB observations where L-BSE has a slightly reduced MM compared to C-BSE.

The tryptic peptide profiles from atypical BSE samples were, as in classical BSE, dominated by T20. Only for sample L2 did it appear that other tryptic peptides (T14 and T15-16) were more abundant. It remains unclear whether this was an analytical artefact due to overall low abundance, or an unusual feature of a specific sample. There did seem to be a recurring difference between the H- and L-BSE samples in the abundance of aglycosyl peptides T15-16 and T18: while T15-16 is of similar or slightly higher abundance than T18 for L-BSE, T15-16 appeared much lower than T18 for H-BSE.

### PrP^res^ profiles following transmission of C-, H- and L-BSE to Tg bov mice

To evaluate PrP^Sc^ profiles following the transmission of C-, H- and L-BSE to Tg bov models, brain samples from transmission studies of UK single source inocula into Tg110 and Tg1896 mice were prepared and analysed (TE_max_ = 2–6 mg). Two individual mouse samples were processed for each inoculum/Tg mouse combination except in one case (H-BSE into Tg1896), where only one sample was available. The concentrations of the N-TAAP and tryptic peptides detected (S5, S6 and S7 Figs) were generally higher compared to the bovine samples, yet different for the different types of BSE. The PrP^res^ yield from the C-BSE infected Tg110 mice was approx. 175 fmol/μl for M1 and 350 fmol/μl for M2 (S5 Fig), 12–20 fold higher than the 10–15 fmol/μl (S3 Fig) obtained from cattle. This increase is not explained by differences in TE_max_, and is large even considering that Tg110 mice overexpress PrP^C^ 8-fold compared to cattle [42]. For H-BSE, the increase in PrP^res^ yield was less extreme: four-fold, from approx. 10 fmol/μl for cattle (S4 Fig) to 40 fmol/μl for Tg bov mice (S6 Fig). For L-BSE, a similar yield of approx. 15 fmol/μl was obtained for Tg110 mice compared to cattle (Figures M9 and M10 in S7 Fig) which was approx. 10-fold higher at 150 fmol/μl in Tg1896 mice (Figures M11 and M12 in S7 Fig).

**Fig 4:**
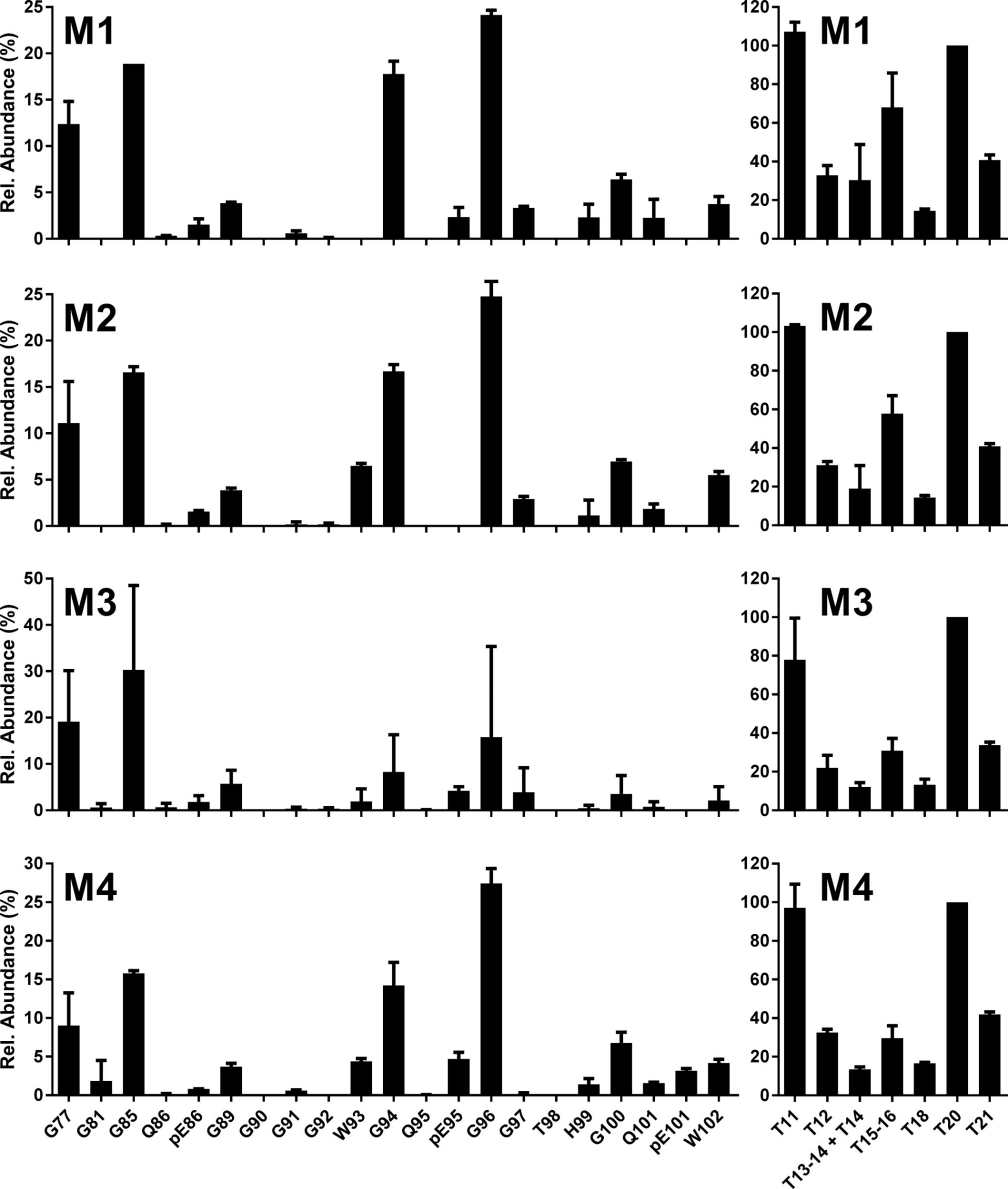
C-BSE into bovinised mice. N-TAAP (left-hand panels) and tryptic peptide profiles (right hand panels) from transgenic mice inoculated with C-BSE from a single UK source. Samples (200–300 mg) were divided into two replicates prior to PK treatment and processed and analysed in parallel, then data were combined to create the profiles. M1, M2: Tg110; M3, M4: Tg1896. TE_max_: M1 = 5.5, M2 = 4.2, M3 = 4.5, M4 = 5.8.

N-TAAPs obtained from C-BSE transmission into either Tg110 or Tg1896 mice showed that cleavage sites G96 and G94 were most abundant, just as observed for bovine C-BSE (Fig 4 and S5 Fig, left-hand panels). Also like bovine C-BSE, cleavage sites G100, pE101 and W102 were detected with an abundance of about 20% of G96 each. Q101, not observed for bovine C-BSE, was also detected at about half the abundance of pE101. The pyroglutamyl form of residue Q95, pE95, was barely detectable for bovine C-BSE but more clearly observed in the Tg bov preparations. Furthermore Tg110 and Tg1896 appeared to differ with regard to the abundance of pE95, which was low for Tg110 but clearly present for Tg1896. In contrast to bovine C-BSE, the further N-terminal sites G85 and G77 were also relatively abundant in the C-BSE Tg mice. G89 was also detected but much less abundant than the neighbouring sites G85 and G94.

**Fig 5:**
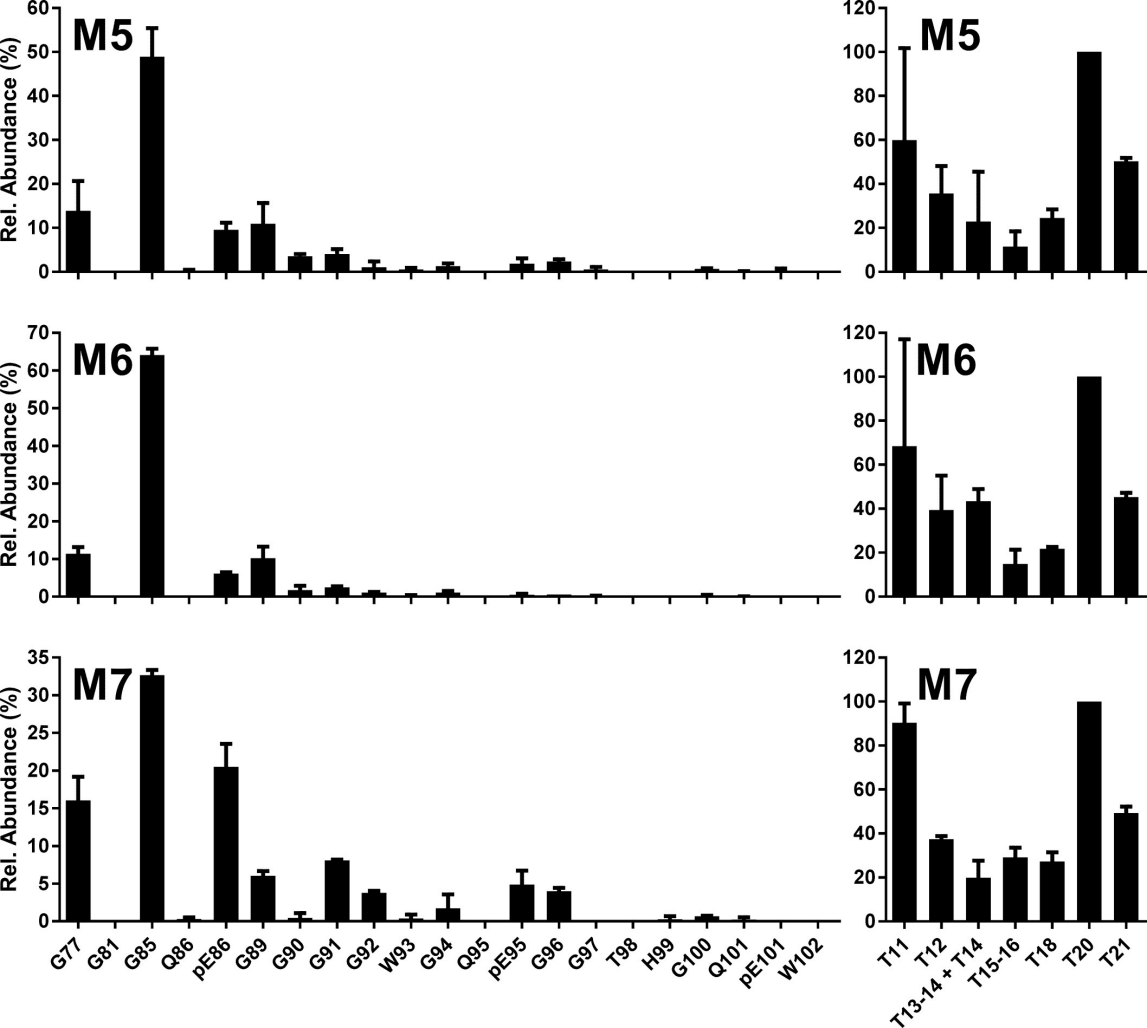
H-BSE into bovinised mice. N-TAAP (left-hand panels) and tryptic peptide profiles (right hand panels) from transgenic mice inoculated with H-BSE from a single UK source. M5, M6: Tg110; M7: Tg1896. Samples (approx. 200 mg each from Tg110 mice, 300 mg from the Tg1896 mouse) were divided into two replicates prior to PK treatment and processed and analysed in parallel, then data were combined to create the profiles. TE_max_: M5 = 3.6, M6 = 3.5, M7 = 5.5.

**Fig 6:**
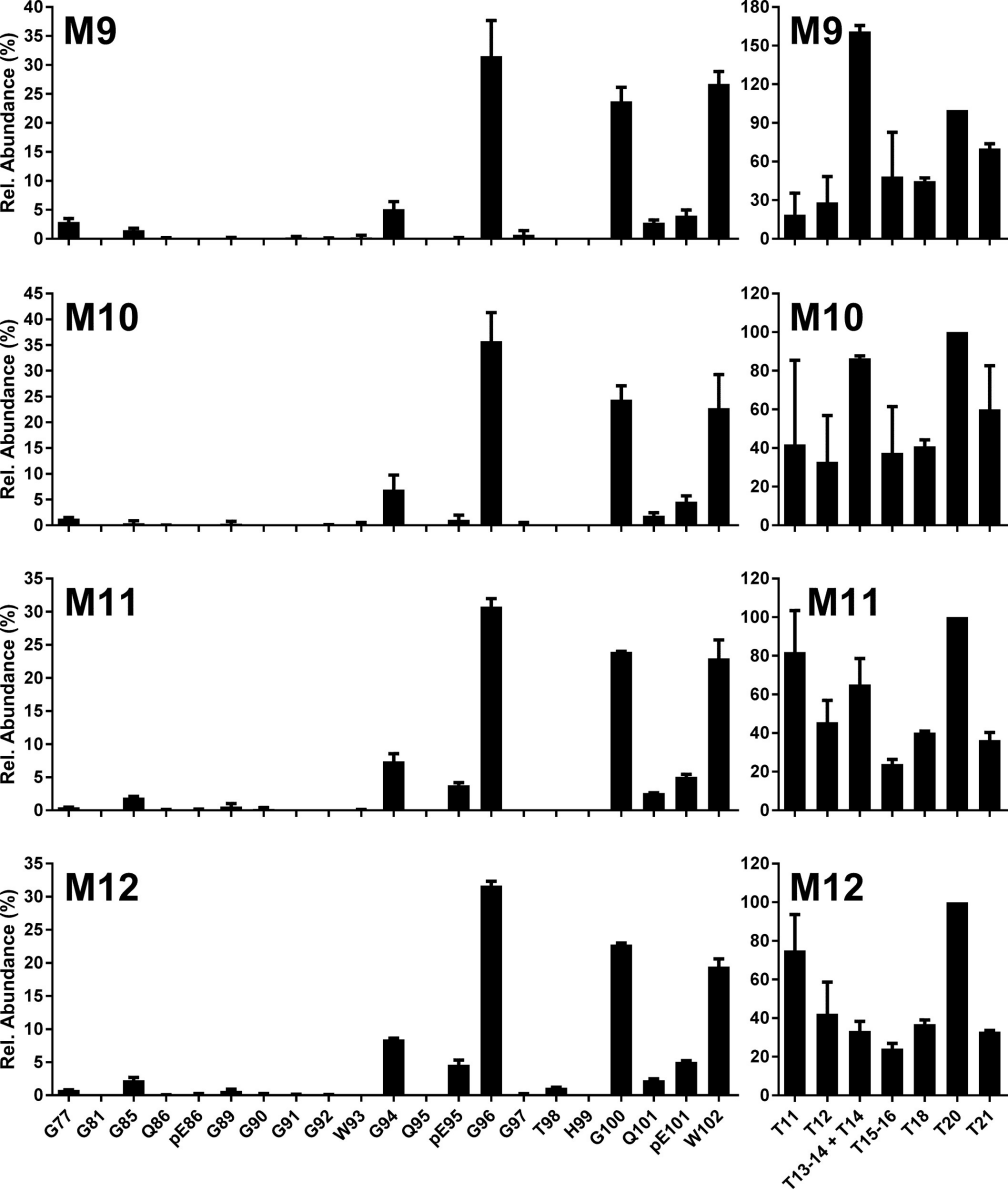
L-BSE into bovinised mice. N-TAAP (left-hand panels) and tryptic peptide profiles (right hand panels) from transgenic mice inoculated with L-BSE from a single UK source. M9, M10: Tg110; M11, M12: Tg1896. Samples (approx. 100mg for Tg110 and 200mg for Tg1896) were divided into two replicates prior to PK treatment and processed and analysed in parallel, then data were combined to create the profiles. TE_max_: M9 = 1.8; M10 = 1.8, M11 = 3.4, M12 = 3.8.

N-TAAPs of H-BSE transmitted to Tg bov mice (Fig 5 and S6 Fig) corresponded to the profiles observed in cattle, where G85 was the most abundant cleavage site followed by G77, pE86 and G89. Cleavage at G94, found to be relatively low for H-BSE in cattle (Fig 3 and S4 Fig) compared to classical scrapie, was also relatively low in the Tg mouse models of H-BSE (Fig 5 and S6 Fig). N-TAAPs for L-BSE in Tg bov mice (Fig 6 and S7 Fig) were consistent with those of L-BSE in cattle (Fig 3 and S4 Fig), with G96 the most abundant, very closely followed by G100 and W102. The relatively low abundance of pE101 in the L-BSE profiles of Tg bov mice was considered to be a minor difference with L-BSE in cattle. pE86, not abundant in either case, appeared to be at a slightly higher level for Tg1896 mice (Fig 6C and 6D) compared to Tg110 mice (Fig 6A and 6B).

The tryptic profiles of the Tg bov mouse line and TSE combinations are consistent, as demonstrated by the small error bars in the relative profiles (Figs 4–6). The high absolute concentrations of the tryptic peptides facilitated their accurate quantification, except for T11, quantification of which is prevented by its relatively low abundance in combination with a high LoQ of 5 fmol/μl.

Differences are evident in absolute and relative abundances of the aglycosyl forms of peptides T15-16 and T18 between the BSE strains and the different Tg mouse lines. For C-BSE in Tg bov mice, a relatively high abundance of the aglycosyl form of T15-16 is observed. For H-BSE and L-BSE in the Tg bov mouse models, T15-16 and T18 are of similar abundance although their abundance compared to T20 appears higher for L-BSE. These differences are analysed further in a section below.

### Changes to L-BSE PrP^res^ in the ovine host are characterised by a broad N-terminal cleavage distribution and C-terminal truncation

**Fig 7:**
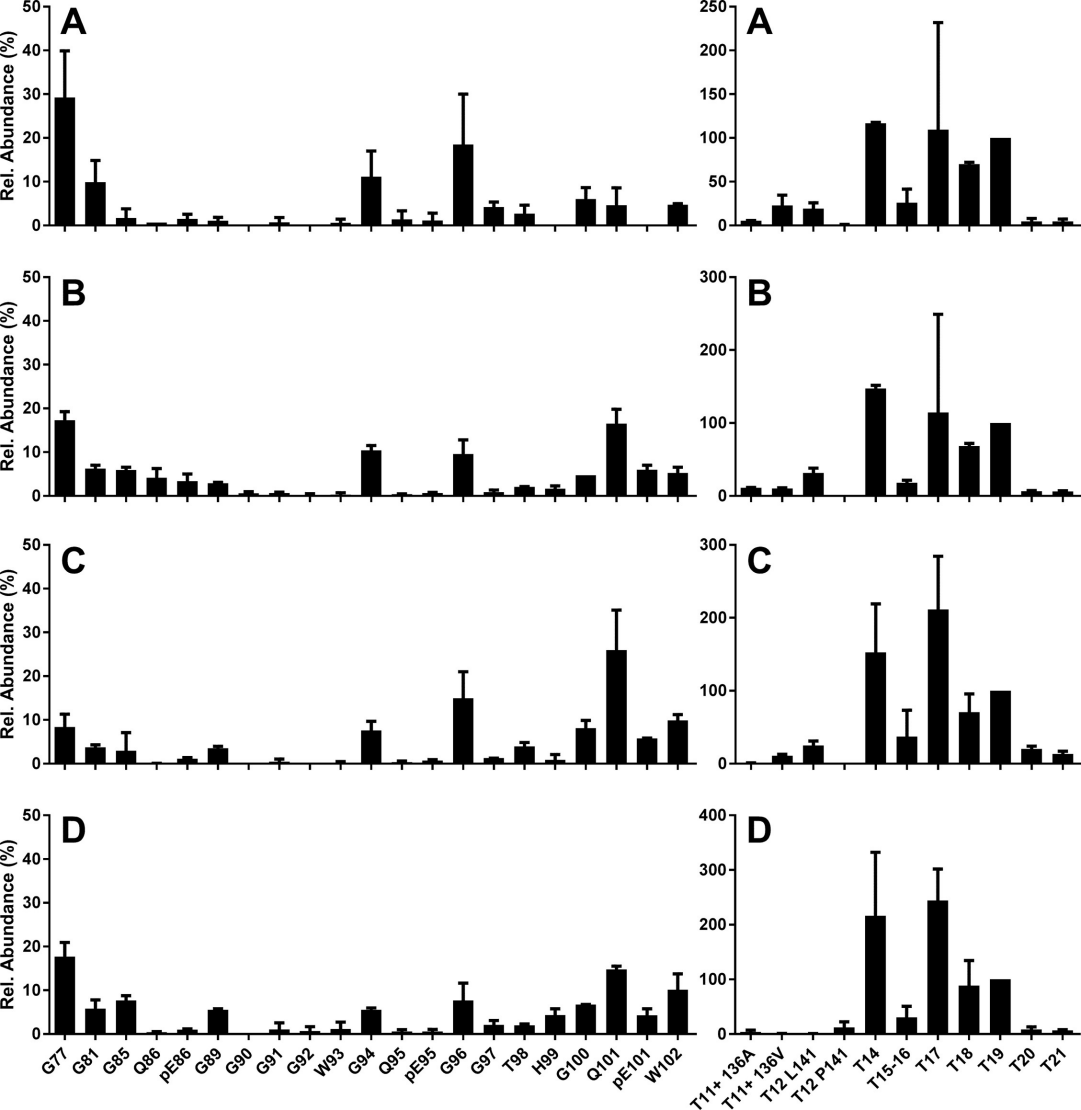
L-BSE into sheep. N-TAAP (left-hand side) and tryptic peptide profiles (right hand side) from sheep inoculated with the same L-BSE source as used in the bovine experimental transmissions, as described in [18]. (A) animal ID 456/11 ALRQ/VRQ, (B) 140/11, ALRQ/VRQ (C) 267/11 VRQ/VRQ (Dorset males) and (D) 457/11 AFRQ/AFRQ (Cheviot). T11+ denotes the summed contributions of T11, T11-12 and T11-13. T12 L141 denotes the T12 variant containing the L141 allele; T12 F141 denotes the T12 variant containing the F141 allele. Samples (approx. 350 mg) were divided into two replicates prior to PK treatment and processed and analysed in parallel, then data combined to create the profiles. TE_max_ = 7mg for each profile.

To gain insight into how bovine atypical BSEs might manifest if transmitted to other species, successful experimental transmissions of L-BSE to sheep have been carried out by other groups [17, 47] as well as at APHA [18], and were found to result in a unique disease phenotype. To better understand these changes at a molecular level, we selected four samples from the APHA ovine L-BSE transmission experiments, in which the same source had been used as for the bovine L-BSE described above, and processed and analysed these using the ovine PrP assay [35]. The resulting relative N-TAAP and tryptic peptide profiles are displayed in Fig 7; corresponding absolute abundances are plotted in S8 Fig. The highest overall abundance was obtained from samples 140/11 and 267/11 (tryptic peptide abundance in the range of 100–300 fmol/μl), followed by 457/11 (50–100 fmol/μl) and 456/11 (10–30 fmol/μl). In WB analyses of these same samples [18], 140/11 and 267/11 displayed intense bands (Fig 4 panel C lanes 3 and 4 in [18]), while 456/11 (Fig 4 panel C lane 5 in [18]) and 457/11 (Fig 4 panel A lane 3 in [18]) were fainter, so our observations in this respect agreed with these analyses. The overall abundances determined for ovine L-BSE corresponded to the relative WB intensities obtained from these animals [18].

The N-TAAP plots show that N-terminal cleavages at G96, G100 and W102, as observed for L-BSE in cattle and Tg bov mice, were accompanied by cleavage at neighbouring sites G94 and Q101 as well as at further N-terminal sites G77, G81, G85 and G89 (Fig 7 and S8 Fig, left hand panels). Moreover, there appears to be no single dominating N-TAAP peptide fragment or set of fragments. The relatively low concentrations of N-TAAP peptides, in the range of 1–10 fmol/μl, compared to tryptic peptide abundance (S8 Fig) or no more than 30% of the total (Fig 7), is a consequence of the broad spread of the cleavage sites across the protease sensitive area of PrP^Sc^.

Absolute and relative tryptic peptide plots are shown in the right-hand panels of S8 Fig and Fig 7, respectively. As the ovine assay included tryptic peptides representing the 136A/V, 141L/F, 154R/H and 171Q/H PRNP genotype variants [48, 49] in sheep (S1 Table), the plots have been expanded to accommodate these peptides. For visual simplicity, abundances of missed cleavage peptides (produced where trypsin has failed to cleave between two amino acid residues it normally targets), that include the same PrP areas, such as T11, T11-12 and T11-13, were added to the same channel. In some cases, tryptic profiles were derived from 136A/V 141L/L 154R/R 172Q/Q animals and therefore contain signal from both A136 and V136 allele forms of tryptic peptides T11, T11-T12 and T11-T13, the sum of which is presented in channels designated “T11+ 136A” and “T11+ 136V” (Fig 7A and 7B and Figures A and B in S8 Fig), while in another case the tryptic profile merely contains peptides corresponding to V136 from a 136V/V 154R/R 172Q/Q genotype animal (Fig 7C and Figure C in S8 Fig). One tryptic profile from a 136A/A 141F/F 154R/R 172Q/Q animal, exclusively shows the T12 variant corresponding the F141 allele (Fig 7D and Figure D in S8 Fig) which is absent from Fig 7A–7C. Since the T11-T12 or T11-T13 peptides corresponding to the F141 allele variant were not included in the ovine assay (S1 Table), peptide abundance in the “T11+ A136” channel was accordingly lower. Also included is tryptic peptide IMER (T19) which, due to the lower hydrophobicity of the bovine variant of T19 (MMER, MEEK index [46] 1.6 as opposed to 5.6 for IMER), was not included in the bovine assay.

The most surprising feature of the ovine L-BSE tryptic profiles in Fig 7 is the comparatively low abundance of T20, which normally dominates PrP^Sc^ tryptic peptide profiles as shown above. C-terminal peptide T21 was of similarly low abundance while the abundance of peptide T19 was of a level similar to T14 and T17. This suggests that the majority of PrP^Sc^ from ovine L-BSE is PK sensitive in the C-terminal area beyond T19. To prevent the reduced abundance of T20 causing unrealistic relative abundances for the other tryptic peptide profiles in Fig 7, we normalised relative to T19, instead.

To further investigate how classical and atypical BSE cause the ovine prion protein to misfold, brain material was prepared and analysed from TgEM16 mice to which C-, H- and L-BSE were transmitted. It was not possible to acquire data from peptide calibration standards added on to these analyses due to technical issues, and therefore quantification of PrP peptides in these samples could not be carried out. A qualitative comparison between the N-TAAP and tryptic peptide chromatograms with those from ovine L- and C-BSE is provided instead (S9 Fig). C-BSE in TgEM16 mice showed the features similar to C-BSE in cattle, but also included the more N-terminal cleavage sites G85 and G89 (Panel A in S9 Fig). T20 dominates the tryptic peptide profiles of C-BSE transmitted to TgEM16 mice (Panel B in S9 Fig). The PrP peptide yield from the H-BSE inoculated TgEM16 mice yield was very low (Panel C in S9 Fig), consistent with the lack of clinical signs and negative WB data from these mice. Nonetheless, some PrP tryptic peptides were detected (Panel D in S9 Fig), possibly due to from high PrP^C^ levels (8-16x) in TgEM16 mice resulting in a certain amount being co-purified with other proteins normally detected in control preparations [35], but without giving rise to the N-TAAP peptides diagnostic of TSEs. N-TAAP and tryptic peptides were detected with good abundance for L-BSE in TgEM16 mice (Panels E and F in S9 Fig), and corresponded to PK cleavage at the sites G94, G96, S100, Q101, pE101, W102, as well as G89, but at relatively low levels compared to classical BSE in TgEM16. T20 was the most abundant (highest) peak for L-BSE in TgEM16 (Panel F in S9 Fig), similar to findings for C-BSE but contrasting with the findings described above for ovine L-BSE. To facilitate a direct comparison between the EM16 L-BSE and ovine L-BSE data, the original N-TAAP and tryptic peptide chromatograms are provided for ovine L-BSE sample 267/11 (Panels G and H in S9 Fig). To underline that the reduced abundance of T20 is uniquely found for L-BSE in the sheep host, N-TAAP and tryptic peptide chromatograms from C-BSE (Panels I and J in S9 Fig) and CH1641 scrapie (Panels K and L in S9 Fig) in a sheep host, are also provided. The latter two are obtained from the same 136 A/A 154 H/H 171 Q/Q samples for which N-TAAPs were published previously [35].

### Aglycosyl peptide analysis

**Fig 8:**
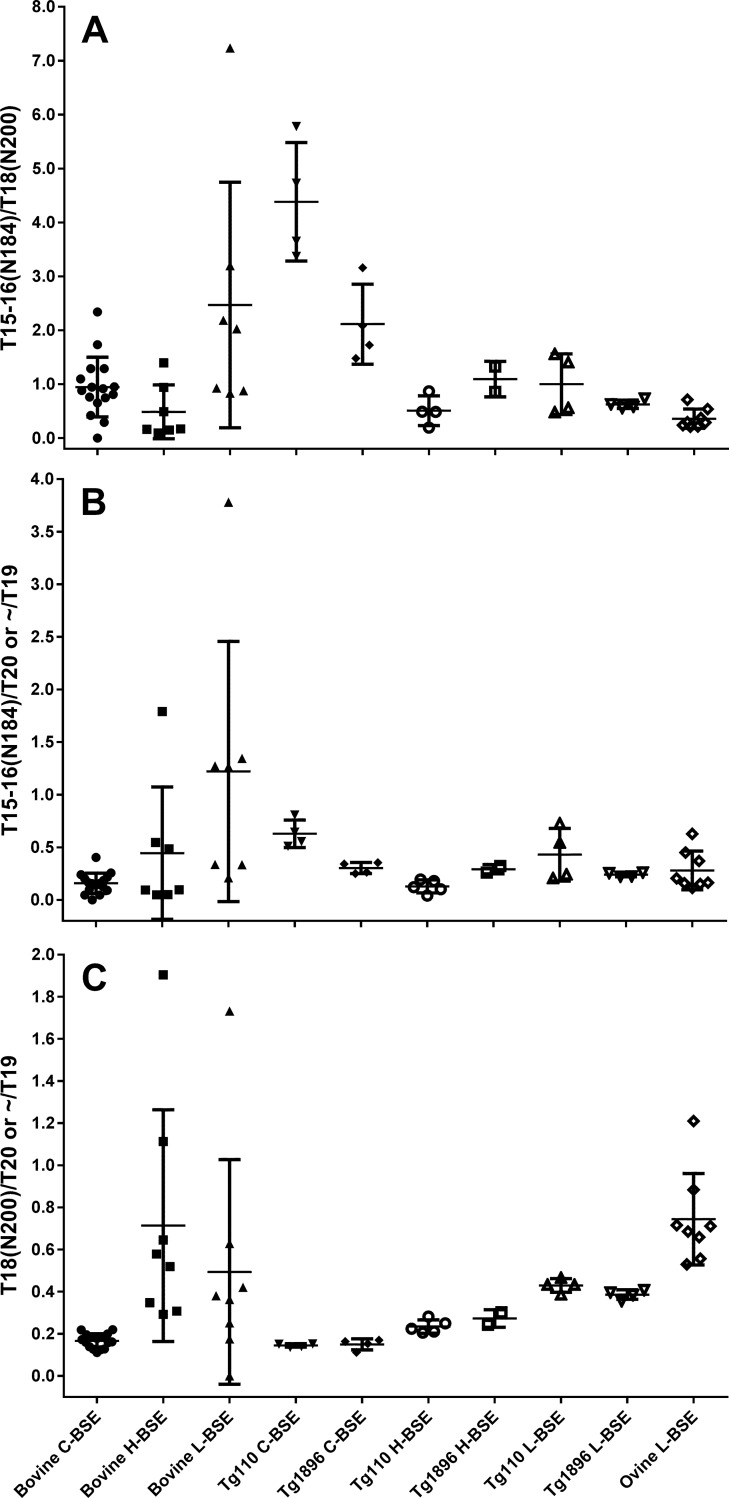
Aglycosyl peptide ratios. Abundance ratios were calculated (A) between the aglycosyl forms of the tryptic peptides T15-16 (Y160-K188) and T18 (G198-K207) which respectively contain glycosylation site N184 and N200, (B) between the aglycosyl form of T15-16 and marker peptide T20 (T19 for ovine L-BSE), and (C) between the aglycosyl form of T18 and marker peptide T20 (T19 for ovine L-BSE). All peptide abundances were normalised to T20, except for ovine L-BSE, where T19 (I208-R211) was used due to the C-terminal cleavage inferred for this TSE and host combination, making it unsuitable as a standard for normalisation.

Glycosylation at N184 and/or N200 is a well-known *in vivo* modification of PrP [25] resulting in a population of four different glycoform groups: the non-glycosylated isoform (A) as well as two types of mono-glycosylated (M1 and M2), and one diglycosylated (D) isoform of PrP. These four glycoforms correspond to the three bands of PrP when detected by WB, as M1 and M2 can not be separated by one-dimensional gel electrophoresis. Differences in MM and glycoforms ratios form the molecular basis for differentiating C-, H- and L-BSE [6, 29]. While differences in WB migration distance between C- and L-BSE are subtle, these TSEs can normally be discriminated taking differences in their glycoform profiles into account: the band corresponding to the D isoform of PrP^Sc^ is much more prominent for bovine C-BSE compared to L-BSE. Plotting the percentages of glycoforms provides a useful visual to display these differences [12, 13, 18, 28, 29, 50]. While the glycosylated forms of the tryptic peptides containing N184 and N200 could not be detected in the MS-based assay, the abundances of the two aglycosyl peptides were routinely determined. Thus their relative ratios to each other and to other PrP^res^ core peptides could be calculated as a proxy for glycoform ratios (Fig 8). In the sections above, differences in aglycosyl peptide abundance between the TSEs in the different hosts were visually identified, but it was not obvious whether these were significant. The ratio between tryptic peptides T15-16 and T18, harbouring the first and second glycosylation site, appeared somewhat variable for the cattle samples (Fig 8A), presumably due to the relatively low general abundance of PrP^res^. By applying a two-tailed t-test and requiring P<0.05, we were able to establish that the differences between bovine L-BSE and bovine C- BSE T15-16/T18 ratios were nonetheless statistically significant (P = 0.0175) (Table A in S4 Table). Similarly, differences in ratios between the aglycosyl peptides and tryptic peptide T20 (Fig 8B and 8C) appeared variable but T15-16/T18 and T15-16/T20 ratios for C-BSE versus L- BSE were significantly different (P = 0.0020 resp. 0.0203) while T18/T20 was also significantly different for C- versus H-BSE (P = 0.0005) (Tables B and C in S4 Table).

Looking at differences between Tg mouse TSEs, where higher PrP^Sc^ yields provided more accurate determinations of aglycosyl peptide abundance, differences in all three ratios were visually clearer (Fig 8A–8C) and also gave rise to lower P-values (S4 Table). Here, T16/T18 was significantly different for 7 out of 12, T16/T20 for 6/12 and T18/T20 for 12/12 comparisons (S4 Table, thickly outlined cells). Thus the ratio between the second glycosylation site and overall protein abundance appeared to be the most sensitive parameter for C-, H- and L-BSE differentiation in Tg bov mice. Moreover, the Tg bov mouse data showed that there were significant differences between the individual mouse lines for the T16/T18 and T16/T20 ratios in C-BSE (p = 0.0141 resp. 0.0036) while differences for H-BSE (p = 0.081 and 0.0216) were less clear-cut and not significant for L-BSE (p = 0.2256 and 0.1817) (Tables A and B in S4 Table).

Both the T16/T18 and T16/T20 ratios were significantly different between C-BSE in cattle versus C-BSE in Tg110 or Tg 1896 mice (P<0.0001, <0.0001, = 0.0023 and = 0.0107), but not between H-BSE or L-BSE in cattle versus the same TSE in Tg mice, while no significant difference was observed in this regard for the T18/T20 ratios (S4 Table, double-outlined cells).

Finally, T16/T18 was significantly different between ovine L-BSE compared to bovine C-BSE (p = 0.0085) but also between bovine and ovine L-BSE (P = 0.0209) and Tg mouse L-BSE (P = 0.0120 for Tg110 and 0.0183 for Tg1896). Given the previously identified C-terminal truncation of PrP^res^ in ovine L-BSE, the tryptic peptide T19 (I208-R211) was used instead of T20 to approximate the ratios between the abundance of the aglycosyl peptides and overall PrP^res^. No significant difference was found between ovine L-BSE T16/T20 and any of the other transmissions, while T18/T20 was significantly different between ovine L-BSE and all except bovine H- and L-BSE (Table C in S4 Table, rightmost column).

Overall, MS-based determination of the relative abundances of aglycosyl peptides has demonstrated that these are significantly different between BSE types, as well as between hosts.

## Discussion

The aims of the present study were (1) to establish the N-terminal amino acid and tryptic peptide profiles of classical and atypical BSE in cattle and identify any differences between them, and (2) to determine any changes in these profiles following transmission of each of the BSE types into Tg bov (Tg110 and Tg1896), and Tg ov (TgEM16) mice, and into sheep, as homologous and heterologous hosts used to determine the stability of these TSEs. The enhanced resolution provided by mass spectrometry based assays improved the confidence with which the stability of PrP^Sc^ conformations associated with these TSEs could be asserted.

### Assay

We expanded our PrP^Sc^ profiling capability by developing a mass spectrometry based assay adapted for bovine PrP, additional to the previously reported ovine assay [35]. The bovine assay allowed determination of N-terminal cleavage sites in the partially protease resistant region of bovine PrP, as well as quantification of tryptic peptides released from the protease resistant region once “melted” by guanidine hydrochloride, since comparison of the relative abundances of tryptic peptides had further potential to reveal differences in PrP^res^ structure(s), particularly regarding the glycosylation sites and C-terminus. However, tryptic PrP^res^ peptides can vary in concentration for multiple reasons such as missed cleavage and post-translational modifications [35]. While all fully tryptic peptides were present at the same level in an intact PrP protein, reasons that that they were detected with different abundances in an mSRM assay include the occurrence of missed cleavages and of *in vivo* or *in vitro* post-translational modifications such as glycosylation, C-terminal truncation [51] and oxidation. These factors have been recognised previously and T20 was found to be the most consistent and to provide the highest level of sensitivity for detection of PrP [52–54]. Presently, only aglycosyl forms of the tryptic peptides containing glycosylation sites, T15-16 for N184, obtainable from A and M2, and T18 for N200, obtainable from A and M1, were included in the assay. Thus T15-16 and T18 were expected to have a lower abundance than T20 (obtainable from all four glycoforms), which is indeed reflected in the data.

Missed cleavage products T11-12 and T11-13 as well as T13-14 (S1 Table) could all be identified at detectable levels, which lead to non-stoichiometric abundance of T11 relative to a mostly fully cleaved peptide such as T20. Even when missed cleavages were taken into account by adding the abundance of its products to that of a completely cleaved peptide, the final concentration did not equate that of other peptides. For instance, the overall T11+ signal (ovine samples) remained low compared to the other tryptic peptides (Fig 7), an observation which is in line with other data for bovine PrP^Sc^ and previous studies into ovine scrapie [35]. Finally, many peptides including T20 contains sites where posttranslational modifications can occur, such as oxidation of methionine (M137, M157, M216) and the reduction and alkylation of cysteine (C182, C217), each process having its own efficiency. Thus the tryptic peptide abundances each represented a fraction of total PrP^res^ and careful interpretation of differences was required, best performed by relative comparisons between samples in assays determined under the same conditions. We have aimed to achieve this by limiting the number of HPLC-MS sequences in which the samples containing bovine PrP were analysed, to two, and carried out these analyses using identical HPLC column settings and calibration conditions. Analysis of the ovine PrP samples naturally required mSRM methods and quantification standards corresponding to ovine instead of bovine PrP peptides and therefore a separate HPLC-MS analysis sequence was used.

### PrP^res^ yields

C-BSE PrP^res^ yields from bovine tissue were generally lower than from the same amount of ovine tissue and more variable from different pieces of tissue. Acquiring mass spectrometry data from a larger amount of bovine tissue sample helped to improve the quality of the chromatograms; nonetheless the relative N-TAAP and tryptic peptide chromatograms remained consistent for smaller and larger amounts of starting material. We established that analysis of an amount of digest corresponding to TE_max_ = 3.5 was a minimum requirement to obtain reliable PrP profiles and an increase in TE_max_ to 7 was achieved by reducing the number of processing replicates per sample from four to two. Increasing TE_max_ further by using larger amounts of starting material or injecting larger sample volumes was considered to promote overloading of the chip-HPLC column resulting in chromatographic displacement, where peptides with lower hydrophobicity would be displaced by more hydrophobic peptides. This would result in peak broadening and general deterioration of results. The relatively low abundance of T21 in one of the experiments where TE_max_ = 10 (Fig 2D–2F) was considered indicative of this. Co-isolated proteins that were not relevant to the present assay, rather than high absolute amounts of PrP^res^, are mainly responsible for this column overloading effect: even in preparations of ovine surveillance cases with good abundance of PrP peptides by mSRM, PrP^res^ was less than 1% of the total amount of protein purified [35], as determined using the spectral counting method to estimate relative protein abundance [55].

PrP^res^ yields from Tg mouse models inoculated with various sources of BSE were considerably higher than from cattle. While this might be attributed to the 8-16x overexpression of PrP^C^ in the Tg mouse models, this relationship is not necessarily linear or proportional. For example, an inverse correlation was found between PrP^C^ overexpression levels and amounts of PrP^Sc^ detected in mice [56], while no particular relationship between the intensity of PrP^res^ bands analysed by WB from bov Tg mouse lines and various PrP^C^ expression levels could be identified [42]. In the present work, the amounts of PrP^res^ detected were also not directly proportional to the overexpression levels and differed depending on the type of BSE. The PrP^res^ yield from Tg bov mice inoculated with C-BSE was approximately twice what was expected based on the level of overexpression, whereas for H-BSE the PrP^res^ yield was less than half what was expected. A striking 10-fold difference in PrP peptide yield was identified between Tg110 and Tg1896 mice infected with L-BSE. These differences were approximately 2-fold for C-BSE and H-BSE. While the use of different H-BSE and L-BSE sources for inoculation of cattle and Tg mice may further complicate the interpretation of these observations, the results indicate that the amount of accumulated PrP^Sc^ is not directly proportional to the onset of clinical signs, and the underlying processes that lead to neuropathology are likely to differ between the TSEs.

### NTAAPs

This is the first time that the PrP^res^ N-TAAPs have been reported for bovine C-, H- and L-BSE, while ovine C-BSE N-TAAPs have been reported previously by our group [33–35]. PK-cleavage sites in bovine C-BSE were dominated by G96 with a small contribution from adjacent sites G94, G100 and W102. Relative abundance plots (S3 Fig) demonstrate that the profiles were consistent between different field cases identified through UK surveillance, and we found no notable differences between these plots and additional data (not shown) obtained from further surveillance cases as well as experimental C-BSE transmissions. While based on a relatively modest 14 samples, the profiles were very similar, further supporting the conclusion that the C-BSE epidemic of cattle was caused by a single strain of infectious agent [57].

Differences in N-TAAPs were identified between C-, H- and L-BSE in cattle, between C-BSE in cattle and C-BSE following transmission to Tg mouse and ovine hosts, and between cattle L-BSE and L-BSE transmitted to ovine hosts. Whereas dissimilarities between the TSEs in the same host were anticipated and are explicable as a direct result of divergent PrP^Sc^ structures, profile changes for a given TSE in a new host were not always expected, and causes can be multifactorial. For C-BSE transmitted to Tg110 mice, PK cleavage appears to have taken place along a somewhat wider stretch of the PrP^Sc^ variably protease sensitive domain and included further N-terminal cleavages such as G89 and G85 (Fig 4 and S5 Fig). The present data, obtained with mass spectrometry as an orthogonal technology and with samples obtained from entirely separate transmission experiments, confirm previous work where a faint signal was detected when monoclonal antibody 12B2 was used to detect BSE-derived PrP^res^ from Tg110 mice, and which was attributed to a minor subpopulation of PrP^Sc^ where the corresponding epitope was preserved [58]. For C-BSE transmitted to ovine hosts, the PK cleavage sites were also found to be more spread out (Fig 7, S8 Fig and [35]), also in keeping with previous findings by WB, where more Group A antibody (P4) reactivity was found for ovine compared to bovine C-BSE [18]. While a relative reduction in PK efficacy may still play a role, also given the much lower PrP^res^ yield from bovine compared to ovine samples described above, bioassay and PMCA studies support the notion of alterations in virulence properties of C-BSE following transmission to sheep [58, 59], indicating that actual changes in PrP^Sc^ structure are likely to underlie the observed spread in PK sites in the ovine host. Equivalent back-transmission studies or protein conversion comparisons to further investigate potential changes to PrP^Sc^ structure also in the case of C-BSE transmissions to Tg110 mice, are unfortunately not available.

H- or L-BSE N-TAAPs in cattle were not found to be particularly different from those in Tg mouse models, but the profile changes for L-BSE transmitted to sheep were dramatically different. The ovine L-BSE N-TAAPs include an unusually wide range of cleavage sites across the partially protease resistant region of PrP^Sc^, and the C-terminus is unusually truncated. These data fit with and underpin previously published molecular studies which reported unusual P4 reactivity while the MM continued to remain below that of C-BSE, contributing to the conclusion that ovine L-BSE presents a unique disease phenotype [18]. However, this is not thought to be the result of a mixture TSEs, as a mixture would lead to a distribution of molecular phenotypes within a challenged group of animals. Instead it is suspected that the broader spectrum is a feature of a single type of PrP^Sc^. By revealing the C-terminal truncation of ovine L-BSE PrP^res^, our studies help to understand the nature of the changes to PrP^Sc^ structure that have taken place. The WB banding pattern or glycoform ratio of PrP^res^ resulting from L-BSE transmission to TgEM16 mice showed remarkable similarity to that resulting from C-BSE transmitted to Tg1896 mice (S1 Fig). It has been reported previously that C-BSE and L-BSE acquire similar PrP^res^ profiles following passage in TgOv mice [12]. However, neither the increased N-terminal cleavage sites further to N-terminus nor the C-terminal truncation stood out in the MS-based data from L-BSE transmitted to TgEM16 mice (Panels E and F in S9 Fig). Since the L-BSE source used to inoculate the sheep host was different from the source used to inoculate the TgEM16 mice, a source-effect is a likely explanation, and since the genotypes of the sheep hosts (ARQ/VRQ, VRQ/VRQ or ARQ/ARQ) are different from the TgEM16 mice (AHQ/AHQ), this is also a potential cause for the observed differences, and further studies would be required to assess the impact of these variables. Nonetheless, more general differences in PrP^Sc^ processing between the two animal species remain a feasible explanation, also in the light of the above-mentioned alterations to C-BSE in sheep versus Tg bovinised mice. This underlines the insights that Tg mouse based transmission models are unlikely to be a wholly equivalent substitute for large animal transmission studies in evaluating TSE transmission risks [60, 61], from a molecular perspective.

The bovine H-BSE N-TAAPs confirm that there must be differences in structure of associated PrP^Sc^ between this TSE and ovine classical scrapie [35], even though they show similar migration and antibody reactivity on WB. Previously, the N-TAAPs from ovine classical scrapie cases were found to either contain a relatively high proportion of cleavage sites between G89 and G94, or an abundant pE95 [35]. Similar marker peptides are not detected for H-BSE (Fig 3 and Figures H3 and H4 in S4 Fig), pointing at differences in structure which are likely to be responsible for the relatively poor transmission of H-BSE to sheep and to the TgEM16 ovinised mouse line.

The presence of the pyroglutamyl N-terminally modified residue 101 (pE101) without similar abundance of Q101 in most preparations suggests that N-terminally truncated forms of PrP^Sc^ were present prior to PK treatment [35]. One possible explanation is that these fragments have arisen through endogenous cleavage processes and may be representative of the C2 fragment [62]. The abundance of these N-terminal residues in samples based on bovine PrP is considerably lower in the C-BSE as well as atypical BSE samples for both cattle and Tg mice, compared to previously reported C-BSE and other ovine samples [35]. This suggests that there are fundamental differences in PrP^Sc^ deposition and truncation for bovine and ovine sequences. L-BSE in Tg bov mouse appears to display a relatively lower amount of Q101 and pE101 compared to L-BSE in cattle.

### Glycosylation

Prion protein parameters that allow discrimination of atypical BSEs from C-BSE by WB include glycosylation ratio [6, 29, 63]. Visually clear differences in glycoforms ratios can be seen in the WBs of the PrP^res^ products following transmission to Tg mice (S1 Fig), and the clearest of these blots would allow for diglycoform and monoglycoform ratios to be determined and plotted. We have shown that the relative abundances of aglycosyl peptides as determined by mass spectrometry are also capable of showing significant differences between BSE types, as well as between hosts.

Differences between glycosylation ratios were not only identified between different TSEs in the same species but also following transmission of this TSE to different species. The most sensitive parameter in this context appeared to be T18/T20, followed by T15-16/T18 (Fig 8). The significant differences between TSEs (S4 Table) were anticipated based on WB glycoforms analysis, but notable changes in glycosylation pattern were also evident between mouse lines studied. Differences in glycosylation between mammals and animals from different classes or phyla such as insects or yeast are well known and differences between mammalian cell lines have also been reported [64]. Therefore differences between PrP^Sc^ glycosylation between cattle and mice would not be unprecedented. Unfortunately not enough is known regarding strain and species-specific glycosylation differences of the various sites, where changes in the monoglycosylated state may be most remarkable. Relatively little is known about preferential occupation of glycosylation sites, which have been found to vary between species and brain regions [65–67], and thus further research is needed. The finding that the Tg bov mouse data showed significant differences between the two individual mouse lines for the T15-16/T18 and T15-16/T20 ratios in C-BSE but not so much for H-BSE (p = 0.081 and 0.0216) and not at all for L-BSE (p = 0.2256 and 0.1817) underscores that certain mouse lines may be better models for differentiation of certain TSEs than others even if they exhibit the same PrP sequence at comparable expression levels. While the meaning of these differences remains to be determined, the results generally suggest that aglycosyl peptide ratios are useful parameters indicating where changes in glycosylation pattern occur. Further work including deglycosylation by PNGase and determination of both diglycosylated and aglycosyl PrP peptides would further improve insight and discriminatory power.

### Wider interpretation

In the present work we have applied the enhanced resolution provided by molecular analysis of PrP^res^ using mass spectrometry to obtain insight into how atypical BSEs are different from other TSEs and to identify conformational changes in PrP^Sc^ following transmission across species barriers. While this approach to characterise prions is not likely to be routinely applied due to its specialist nature, it complements methods such as refined epitope mapping to help understand atypical prions at the molecular level without being sensitive to species-related differences in antibody specificity. We identified differences between the high resolution molecular profiles from both L-BSE and H-BSE with those from other TSEs studied to date. Following transmission of C-, H- and L-BSE into Tg mouse lines and into sheep, various changes, some more subtle than others, have been detected: extension of cleavage sites to the N-terminus as well as C-terminal truncation, and differences in glycosylation ratios that were significant between TSE strains as well as animal species or lines.

An improved understanding of where prion structures change following transmission may provide improved understanding of risk of certain TSEs to the food chain. There is ongoing debate regarding the relationship between PrP^Sc^ size, stability and incubation time. For example long disease incubation times have been associated with more stable PrP^Sc^ structures in some studies [27] and with less stable PrP^Sc^ in others [68]. It could be argued that the more structural changes that can occur in a prion population following transmission, the more potential the associated TSE has to cross species barriers. However, more work is needed to understand what type of structural changes, for instance those that result in longer or shorter PK resistant domains, bring about which level of risk. The use of the MS-based PrP^res^ assay with its enhanced resolution, increases our insight into these structural changes. The apparent changeability of the structures of the associated PrP^Sc^ molecules following transmission to sheep underlines the potential risk to the food chain posed by these TSEs. Nonetheless, establishing a direct relationship between PrP^Sc^ conformation and zoonotic risk is complex, and maintenance of current precautionary food chain security measures remains advisable.

## Supporting information

S1 Fig Western blots of C-, H- and L-BSE transmitted to transgenic mouse lines Tg110, Tg1896 and TgEM16. Animal ID/Sample references as in Table 1. Lanes for samples not relevant to the present work have been edited out. Where blots for sample used in MS studies were not available, a WB from a littermate was included, indicated by an additional: M7L: littermate of M7, M11L: littermate of M11, M18L: littermate of M18. Scr = ovine classical scrapie positive control BSE = bovine classical BSE positive control. All blots were run using 12% BisTris gels and Magic Mark XP molecular mass markers (Thermo Fisher) were used.
(PDF)

S2 Fig Ovine PrP protein sequence (136A/154R/171Q) indicating N-TAAP region and tryptic peptides used in the mass spectrometry-based assay (also refer to S1 and S2 Tables). Arrowheads indicate trypsin cleavage sites. Amino acid residues in bold indicate interspecies polymorphisms in comparison with the bovine sequence. For polymorphisms R154H or Q171R, tryptic peptide numbering is maintained.
(PDF)

S3 Fig Absolute N-TAAP (left-hand panels) and tryptic peptide profiles (right hand panels) of natural bovine classical BSE cases from the UK. Case references: (A,D) 06/06990, (B,E) 1643/97, (C,F) 1339/96. Samples (A-C) were prepared using 350 mg, (D-F) using 1000 mg starting material, divided in four prior to PK treatment, and processed and analysed in parallel, giving TE_max_ of 3.5 and 7 mg, resp., then data were combined to create the profiles. Where error bars exceeded the maximum of the y-axis range displayed, they were clipped by the software and drawn in manually. Peptide numbering is as detailed in Fig 1, and in S1 and S2 Tables.
(PDF)

S4 Fig Bovine atypical BSE profiles. Absolute abundances N-TAAP (left-hand panels) and tryptic peptide profiles (right hand panels). H3, H4: H-BSE primary passage cases [37] L2, L4: L-BSE primary passage cases [37]; L6: L-BSE secondary passage case [38]. Samples (350 mg) were divided into two replicates prior to PK treatment and processed and analysed in parallel (TE_max_ = 7 mg), then data combined to create the profiles. Where error bars exceeded the maximum of the y-axis range displayed, they were clipped by the software and drawn in manually.
(PDF)

S5 Fig C-BSE in bovinised mice. N-TAAP (left-hand panels) and tryptic peptide profiles (right hand panels) from transgenic mice inoculated with C-BSE from a single UK source. Samples (200–300 mg) were divided into two replicates prior to PK treatment and processed and analysed in parallel, then data were combined to create the profiles. M1, M2: Tg110; M3, M4: Tg1896. TE_max_ M1 = 5.5, M2 = 4.2, M3 = 4.5, M4 = 5.8.
(PDF)

S6 Fig H-BSE into bovinised mice. N-TAAP (left-hand panels) and tryptic peptide profiles (right hand panels) from transgenic mice inoculated with H-BSE from a single UK source. M5, M6: Tg110; M7: Tg1896. Samples (approx. 200 mg each from Tg110 mice, 300 mg from the Tg1896 mouse) were divided into two replicates prior to PK treatment and processed and analysed in parallel, then data were combined to create the profiles. TE_max_: M5 = 3.6, M6 = 3.5, M7 = 5.5.
(PDF)

S7 Fig L-BSE profiles obtained from individual bovinised mice. Absolute abundance N-TAAP (left-hand panels) and tryptic peptide profiles (right hand panels) from transgenic mice inoculated with L-BSE from a single UK source. M9, M10: Tg110; M11, M12: Tg1896. Samples (approx. 100mg for Tg110 and 200mg for Tg1896) were divided into two replicates prior to PK treatment and processed and analysed in parallel, then data were combined to create the profiles. TE_max_: M9 = 1.8; M10 = 1.8, M11 = 3.4, M12 = 3.8.
(PDF)

S8 Fig L-BSE into sheep. Absolute abundance N-TAAP (left-hand side) and tryptic peptide profiles (right hand side) from sheep inoculated with the same L-BSE source as used in the bovine experimental transmissions, as described in [19]. (A) animal ID 456/11 ALRQ/VRQ, (B) 140/11, ALRQ/VRQ (C) 267/11 VRQ/VRQ (Dorset males) and (D) 457/11 AFRQ/AFRQ (Cheviot). T11+ denotes the summed contributions of T11, T11-12 and T11-13. T12 L141 denotes the T12 variant containing the L141 allele; T12 F141 denotes the T12 variant containing the F141 allele. Samples (approx. 350 mg) were divided into two replicates prior to PK treatment and processed and analysed in parallel, then data combined to create the profiles. TE_max_ = 7mg for each profile.
(PDF)

S9 Fig (A, B) N-TAAP and tryptic peptide chromatograms of C-BSE in a TgEM16 mouse (M13). (C, D) N-TAAP and tryptic peptide chromatograms of H-BSE in a TgEM16 mouse (M15). (E, F) N-TAAP and tryptic peptide chromatograms of L-BSE in a TgEM16 mouse (M17). (G, H) N-TAAP and tryptic peptide chromatograms of L-BSE in an ARQ/VRQ sheep (267/11). (I, J) N-TAAP and tryptic peptide chromatograms of C-BSE in an AHQ/AHQ sheep (822/05). (K, L) N-TAAP and tryptic peptide chromatograms from CH1641 scrapie in an AHQ/AHQ sheep (851/05).
(PDF)

S1 Table Bovine and ovine PrP tryptic peptides and how these have been included in the assays and in plots. Amino acid residues in **bold**: bovine/ovine interspecies polymorphisms; underlined: ovine intraspecies polymorphisms. pE denotes a pyroglutamyl N-terminal amino acid residue. C^ep^ denotes the ethylpyridyl alkylated product of cysteine; N* indicates an N-glycosylation site.
(PDF)

S2 Table Bovine and ovine PrP semi-tryptic peptides used for N-terminal amino acid profiling. The semi-tryptic peptides are the product of N-terminal cleavage by PK of tryptic peptide T7-T9, a sequence which includes two KP missed cleavages. Amino acid residues in bold: bovine/ovine interspecies polymorphisms. pE denotes a pyroglutamyl N-terminal amino acid residue.
(PDF)

S3 Table Average (av) molecular mass, precursor and product ions used in assay, and limits of detection (LoDs) and lower limits of quantification (LoQs) for bovine PrP peptides included in the assay. The LoD was defined as the concentration above which peptides could be detected with a signal-to-noise ratio larger than 3. The LlOQ was defined as the concentration above which the mean concentration determined for three QC standards was within 20% of the expected concentration. ND = not determined (returned values were outside required range). The equivalent values for ovine peptides have been published previously [35].
(PDF)

S4 Table P-values for two-tailed t-test for significant differences between glycoforms ratios. **Bold**: significant difference (P< 0.05), ***Bold italic*** (P<0.01). Outlined areas as referred to in text.
    Table A: P-values for differences between BSE types: T16/T18
    Table B: P-values for differences between BSE types: T16/T20
    Table C: P-values for differences between BSE types: T18/T20
(PDF)

S1 File Data underlying Figs 2–7 (Graphpad Prism format). (ZIP)

S2 File Data underlying Fig 8 (Graphpad Prism format). (ZIP)

S3 File Data underlying S3–S8 Figs (Graphpad Prism format). (ZIP)

## References

[1] Wells GA, Scott AC, Johnson CT, Gunning RF, Hancock RD, Jeffrey M, et al A novel progressive spongiform encephalopathy in cattle. Vet Rec. 1987;121(18):419–20. Epub 1987/10/31. . 3424605 10.1136/vr.121.18.419

[2] Hope J, Reekie LJ, Hunter N, Multhaup G, Beyreuther K, White H, et al Fibrils from brains of cows with new cattle disease contain scrapie-associated protein. Nature. 1988;336(6197):390–2. 10.1038/336390a0 . 2904126

[3] Wilesmith JW, Wells GA, Cranwell MP, Ryan JB. Bovine spongiform encephalopathy: epidemiological studies. Vet Rec. 1988;123(25):638–44. . 3218047

[4] Buschmann A, Gretzschel A, Biacabe AG, Schiebel K, Corona C, Hoffmann C, et al Atypical BSE in Germany—proof of transmissibility and biochemical characterization. Vet Microbiol. 2006;117(2–4):103–16. Epub 2006/08/19. 10.1016/j.vetmic.2006.06.016 . 16916588

[5] Biacabe AG, Laplanche JL, Ryder S, Baron T. Distinct molecular phenotypes in bovine prion diseases. EMBO Rep. 2004;5(1):110–5. Epub 2004/01/08. 10.1038/sj.embor.7400054 ; PubMed Central PMCID: PMCPmc1298965. 14710195 PMC1298965

[6] Casalone C, Zanusso G, Acutis P, Ferrari S, Capucci L, Tagliavini F, et al Identification of a second bovine amyloidotic spongiform encephalopathy: molecular similarities with sporadic Creutzfeldt-Jakob disease. Proc Natl Acad Sci U S A. 2004;101(9):3065–70. Epub 2004/02/19. 10.1073/pnas.0305777101 ; PubMed Central PMCID: PMCPmc365745. 14970340 PMC365745

[7] Jacobs JG, Sauer M, van Keulen LJ, Tang Y, Bossers A, Langeveld JP. Differentiation of ruminant transmissible spongiform encephalopathy isolate types, including bovine spongiform encephalopathy and CH1641 scrapie. J Gen Virol. 2011;92(Pt 1):222–32. Epub 2010/10/15. doi: vir.0.026153–0 [pii] 10.1099/vir.0.026153-0 . 20943889

[8] OIE. Number of reported cases of bovine spongiform encephalopathy (BSE) in farmed cattle worldwide (excluding the United Kingdom) 2018. Available from: http://www.oie.int/animal-health-in-the-world/bse-specific-data/number-of-reported-cases-worldwide-excluding-the-united-kingdom/.

[9] Collinge J, Sidle KC, Meads J, Ironside J, Hill AF. Molecular analysis of prion strain variation and the aetiology of 'new variant' CJD. Nature. 1996;383(6602):685–90. PubMed PMID: Neurogenetics Unit, Department of Biochemistry and Molecular Genetics, Imperial College School of Medicine at St. Mary's, London, UK. j.collinge@ic.ac.uk 10.1038/383685a0 8878476

[10] Hill AF, Desbruslais M, Joiner S, Sidle KC, Gowland I, Collinge J, et al The same prion strain causes vCJD and BSE. Nature. 1997;389(6650):448–50, 526. Epub 1997/10/23 22:27. 10.1038/38925 . 9333232

[11] Bruce ME, Will RG, Ironside JW, McConnell I, Drummond D, Suttie A, et al Transmissions to mice indicate that 'new variant' CJD is caused by the BSE agent. Nature. 1997;389(6650):498–501. Epub 1997/10/23 22:27. 10.1038/39057 . 9333239

[12] Beringue V, Andreoletti O, Le Dur A, Essalmani R, Vilotte JL, Lacroux C, et al A bovine prion acquires an epidemic bovine spongiform encephalopathy strain-like phenotype on interspecies transmission. J Neurosci. 2007;27(26):6965–71. Epub 2007/06/29. 10.1523/JNEUROSCI.0693-07.2007 . 17596445 PMC6672218

[13] Beringue V, Bencsik A, Le Dur A, Reine F, Lai TL, Chenais N, et al Isolation from cattle of a prion strain distinct from that causing bovine spongiform encephalopathy. PLoS Pathog. 2006;2(10):e112 Epub 2006/10/24. 10.1371/journal.ppat.0020112 ; PubMed Central PMCID: PMCPmc1617128. 17054396 PMC1617128

[14] Béringue V, Herzog L, Reine F, Le Dur A, Casalone C, Vilotte J-L, et al Transmission of Atypical Bovine Prions to Mice Transgenic for Human Prion Protein. Emerging Infectious Diseases. 2008;14(12):1898–901. doi: 10.3201/eid1412.080941. 10.3201/eid1412.080941 19046515 PMC2634647

[15] Capobianco R, Casalone C, Suardi S, Mangieri M, Miccolo C, Limido L, et al Conversion of the BASE prion strain into the BSE strain: the origin of BSE? PLoS Pathog. 2007;3(3):e31 Epub 2007/03/14. 10.1371/journal.ppat.0030031 ; PubMed Central PMCID: PMCPmc1817656. 17352534 PMC1817656

[16] Torres JM, Andreoletti O, Lacroux C, Prieto I, Lorenzo P, Larska M, et al Classical bovine spongiform encephalopathy by transmission of H-type prion in homologous prion protein context. Emerg Infect Dis. 2011;17(9):1636–44. Epub 2011/09/06. 10.3201/eid1709.101403 ; PubMed Central PMCID: PMCPmc3322056. 21888788 PMC3322056

[17] Nicot S, Bencsik A, Migliore S, Canal D, Leboidre M, Agrimi U, et al L-type bovine spongiform encephalopathy in genetically susceptible and resistant sheep: changes in prion strain or phenotypic plasticity of the disease-associated prion protein? J Infect Dis. 2014;209(6):950–9. Epub 2013/11/13. 10.1093/infdis/jit596 . 24218507

[18] Simmons MM, Chaplin MJ, Konold T, Casalone C, Beck KE, Thorne L, et al L-BSE experimentally transmitted to sheep presents as a unique disease phenotype. Vet Res. 2016;47(1):112 Epub 2016/11/09. 10.1186/s13567-016-0394-1 ; PubMed Central PMCID: PMCPmc5101820. 27825366 PMC5101820

[19] Comoy EE, Casalone C, Lescoutra-Etchegaray N, Zanusso G, Freire S, Marce D, et al Atypical BSE (BASE) transmitted from asymptomatic aging cattle to a primate. PLoS One. 2008;3(8):e3017 Epub 2008/08/21. 10.1371/journal.pone.0003017 ; PubMed Central PMCID: PMCPmc2515088. 18714385 PMC2515088

[20] Levavasseur E, Biacabe AG, Comoy E, Culeux A, Grznarova K, Privat N, et al Detection and partial discrimination of atypical and classical bovine spongiform encephalopathies in cattle and primates using real-time quaking-induced conversion assay. PLoS One. 2017;12(2):e0172428 Epub 2017/02/24. 10.1371/journal.pone.0172428 ; PubMed Central PMCID: PMCPmc5322914. 28231300 PMC5322914

[21] Mestre-Francés N, Nicot S, Rouland S, Biacabe AG, Quadrio I, Perret-Liaudet A, et al Oral Transmission of L-type Bovine Spongiform Encephalopathy in Primate Model. Emerg Infect Dis. 2012;18(1):142–5. 10.3201/eid1801.111092 ; PubMed Central PMCID: PMCPmc3310119. 22261009 PMC3310119

[22] Bencsik A, Leboidre M, Debeer S, Aufauvre C, Baron T. Unique properties of the classical bovine spongiform encephalopathy strain and its emergence from H-type bovine spongiform encephalopathy substantiated by VM transmission studies. J Neuropathol Exp Neurol. 2013;72(3):211–8. 10.1097/NEN.0b013e318285c7f9 . 23399901

[23] Requena JR, Kristensson K, Korth C, Zurzolo C, Simmons M, Aguilar-Calvo P, et al The Priority position paper: Protecting Europe's food chain from prions. Prion. 2016;10(3):165–81. Epub 2016/05/26. 10.1080/19336896.2016.1175801 ; PubMed Central PMCID: PMCPmc4981192. 27220820 PMC4981192

[24] Telling GC, Parchi P, DeArmond SJ, Cortelli P, Montagna P, Gabizon R, et al Evidence for the conformation of the pathologic isoform of the prion protein enciphering and propagating prion diversity. Science. 1996;274(5295):2079–82. Epub 1996/12/20. . 8953038 10.1126/science.274.5295.2079

[25] Prusiner SB. Molecular biology of prion diseases. Science. 1991;252(5012):1515–22. PubMed PMID: Department of Neurology, University of California, San Francisco 94143. 1675487 10.1126/science.1675487

[26] Soto C. Prion Hypothesis: The end of the Controversy? Trends in biochemical sciences. 2011;36(3):151–8. 10.1016/j.tibs.2010.11.001 PubMed PMID: PMC3056934. 21130657 PMC3056934

[27] Peretz D, Williamson RA, Legname G, Matsunaga Y, Vergara J, Burton DR, et al A change in the conformation of prions accompanies the emergence of a new prion strain. Neuron. 2002;34(6):921–32. 12086640 10.1016/s0896-6273(02)00726-2

[28] Biacabe AG, Jacobs JG, Bencsik A, Langeveld JP, Baron TG. H-type bovine spongiform encephalopathy: complex molecular features and similarities with human prion diseases. Prion. 2007;1(1):61–8. Epub 2007/01/01. ; PubMed Central PMCID: PMCPmc2633710. 19164888 10.4161/pri.1.1.3828 PMC2633710

[29] Jacobs JG, Langeveld JP, Biacabe AG, Acutis PL, Polak MP, Gavier-Widen D, et al Molecular discrimination of atypical bovine spongiform encephalopathy strains from a geographical region spanning a wide area in europe. J Clin Microbiol. 2007;45(6):1821–9. PubMed PMID: Department of Bacteriology and TSEs, Central Institute for Animal Disease Control (CIDC-Lelystad), 8203 AA 2004, Lelystad. The Netherlands. jan.langeveld@wur.nl 10.1128/JCM.00160-07 17442800 PMC1933055

[30] Chen SG, Zou W, Parchi P, Gambetti P. PrP^Sc^ typing by N-terminal sequencing and mass spectrometry. Archives of Virology (Suppl). 2000;16:209–16. 11214924

[31] Sajnani G, Pastrana MA, Dynin I, Onisko B, Requena JR. Scrapie prion protein structural constraints obtained by limited proteolysis and mass spectrometry. J Mol Biol. 2008;382(1):88–98. Epub 2008/07/16. doi: S0022-2836(08)00797-3 [pii] 10.1016/j.jmb.2008.06.070 . 18621059

[32] Howells LC, Anderson S, Coldham NG, Sauer MJ. Transmissible spongiform encephalopathy strain-associated diversity of N-terminal proteinase K cleavage sites of PrP(Sc) from scrapie-infected and bovine spongiform encephalopathy-infected mice. Biomarkers. 2008;13(4):393–412. Epub 2008/05/20. doi: 793156474 [pii] 10.1080/13547500801903719 . 18484354

[33] Gielbert A, Davis LA, Sayers AR, Hope J, Gill AC, Sauer MJ. High-resolution differentiation of transmissible spongiform encephalopathy strains by quantitative N-terminal amino acid profiling (N-TAAP) of PK-digested abnormal prion protein. J Mass Spectrom. 2009;44(3):384–96. Epub 2008/12/05. 10.1002/jms.1516 . 19053160

[34] Gielbert A, Davis LA, Sayers AR, Tang Y, Hope J, Sauer MJ. Quantitative profiling of PrP peptides by high-performance liquid chromatography mass spectrometry to investigate the diversity of prions. Anal Biochem. 2013;436(1):36–44. 10.1016/j.ab.2013.01.015 . 23357236

[35] Gielbert A, Thorne JK, Hope J. Pyroglutamyl-N-terminal prion protein fragments in sheep brain following the development of transmissible spongiform encephalopathies. Frontiers in molecular biosciences. 2015;2:7 10.3389/fmolb.2015.00007 ; PubMed Central PMCID: PMC4429639. 25988175 PMC4429639

[36] Arnold ME, Ryan JB, Konold T, Simmons MM, Spencer YI, Wear A, et al Estimating the temporal relationship between PrPSc detection and incubation period in experimental bovine spongiform encephalopathy of cattle. J Gen Virol. 2007;88(Pt 11):3198–208. 10.1099/vir.0.82987-0 . 17947547

[37] Konold T, Bone GE, Clifford D, Chaplin MJ, Cawthraw S, Stack MJ, et al Experimental H-type and L-type bovine spongiform encephalopathy in cattle: observation of two clinical syndromes and diagnostic challenges. BMC Vet Res. 2012;8:22 Epub 2012/03/10. 10.1186/1746-6148-8-22 ; PubMed Central PMCID: PMCPmc3378435. 22401036 PMC3378435

[38] Konold T, Phelan LJ, Clifford D, Chaplin MJ, Cawthraw S, Stack MJ, et al The pathological and molecular but not clinical phenotypes are maintained after second passage of experimental atypical bovine spongiform encephalopathy in cattle. BMC Vet Res. 2014;10:243 Epub 2014/10/03. 10.1186/s12917-014-0243-2 ; PubMed Central PMCID: PMCPmc4190426. 25274502 PMC4190426

[39] Jeffrey M, Witz JP, Martin S, Hawkins SAC, Bellworthy SJ, Dexter GE, et al Dynamics of the natural transmission of bovine spongiform encephalopathy within an intensively managed sheep flock. Veterinary Research. 2015;46:126 10.1186/s13567-015-0269-x PubMed PMID: PMC4625529. 26511838 PMC4625529

[40] Stack MJ, Focosi-Snyman R, Cawthraw S, Davis L, Chaplin MJ, Burke PJ. Third atypical BSE case in Great Britain with an H-type molecular profile. Vet Rec. 2009;165(20):605–6. . 19915195 10.1136/vr.165.20.605-c

[41] Stack MJ, Chaplin MJ, Davis LA, Everitt S, Simmons MM, Windl O, et al Four BSE cases with an L-BSE molecular profile in cattle from Great Britain. Vet Rec. 2013;172(3):70 Epub 2012/12/20. 10.1136/vr.101158 . 23249774

[42] Castilla J, Gutierrez Adan A, Brun A, Pintado B, Ramirez MA, Parra B, et al Early detection of PrPres in BSE-infected bovine PrP transgenic mice. Arch Virol. 2003;148(4):677–91. Epub 2003/03/29. 10.1007/s00705-002-0958-4 . 12664293

[43] Preparation of more sensitive bioassay models for the improved detection, differentiation & diagnosis of BSE—SE1753. London: Department for Environment, Food and Rural Affairs, 2009.

[44] Griffiths PC, Plater JM, Chave A, Jayasena D, Tout AC, Rice PB, et al Overexpression of chimaeric murine/ovine PrP (A136H154Q171) in transgenic mice facilitates transmission and differentiation of ruminant prions. J Gen Virol. 2013;94(Pt 11):2577–86 10.1099/vir.0.051581-0 . 23761404

[45] Corda E, Thorne L, Beck KE, Lockey R, Green RB, Vickery CM, et al Ability of wild type mouse bioassay to detect bovine spongiform encephalopathy (BSE) in the presence of excess scrapie. Acta neuropathologica communications. 2015;3:21 10.1186/s40478-015-0194-2 ; PubMed Central PMCID: PMCPMC4382846. 25853789 PMC4382846

[46] Meek JL. Prediction of peptide retention times in high-pressure liquid chromatography on the basis of amino acid composition. Proc Natl Acad Sci USA. 1980;77:1632–6. 6929513 10.1073/pnas.77.3.1632 PMC348551

[47] Okada H, Masujin K, Miyazawa K, Yokoyama T. Acquired transmissibility of sheep-passaged L-type bovine spongiform encephalopathy prion to wild-type mice. Veterinary Research. 2015;46(1):81 10.1186/s13567-015-0211-2 PubMed PMID: PMC4499898. 26169916 PMC4499898

[48] Belt PB, Muileman IH, Schreuder BE, Bos-de Ruijter J, Gielkens AL, Smits MA. Identification of five allelic variants of the sheep PrP gene and their association with natural scrapie. J Gen Virol. 1995;76 (Pt 3):509–17. Epub 1995/03/01. 10.1099/0022-1317-76-3-509 . 7897344

[49] Saunders GC, Cawthraw S, Mountjoy SJ, Hope J, Windl O. PrP genotypes of atypical scrapie cases in Great Britain. J Gen Virol. 2006;87(Pt 11):3141–9. Epub 2006/10/13. 10.1099/vir.0.81779-0 . 17030846

[50] Priemer G, Balkema-Buschmann A, Hills B, Groschup MH. Biochemical Characteristics and PrP(Sc) Distribution Pattern in the Brains of Cattle Experimentally Challenged with H-type and L-type Atypical BSE. PLoS One. 2013;8(6):e67599 Epub 2013/06/28. 10.1371/journal.pone.0067599 ; PubMed Central PMCID: PMCPmc3689710. 23805320 PMC3689710

[51] Stahl N, Baldwin MA, Burlingame AL, Prusiner SB. Identification of glycoinositol phospholipid linked and truncated forms of the scrapie prion protein. Biochemistry. 1990;29(38):8879–84. PubMed PMID: Department of Neurology, University of California, San Francisco 94143. 1980209 10.1021/bi00490a001

[52] Onisko B, Dynin I, Requena JR, Silva CJ, Erickson M, Carter JM. Mass spectrometric detection of attomole amounts of the prion protein by nanoLC/MS/MS. J Am Soc Mass Spectrom. 2007;18(6):1070–9. PubMed PMID: Western Regional Research Center, United States Department of Agriculture, Albany, California, USA. bonisko@pw.usda.gov 10.1016/j.jasms.2007.03.009 17446085

[53] Onisko BC, Silva CJ, Dynin I, Erickson M, Vensel WH, Hnasko R, et al Sensitive, preclinical detection of prions in brain by nanospray liquid chromatography/tandem mass spectrometry. Rapid Commun Mass Spectrom. 2007;21(24):4023–6. Epub 2007/11/15. 10.1002/rcm.3310 . 18000838

[54] Silva CJ, Onisko BC, Dynin I, Erickson ML, Requena JR, Carter JM. Utility of mass spectrometry in the diagnosis of prion diseases. Anal Chem. 2011;83(5):1609–15. Epub 2011/02/04. 10.1021/ac102527w . 21288014

[55] Tang Y, Underwood A, Gielbert A, Woodward MJ, Petrovska L. Metaproteomics analysis reveals the adaptation process for the chicken gut microbiota. Appl Environ Microbiol. 2014;80(2):478–85. 10.1128/AEM.02472-13 ; PubMed Central PMCID: PMC3911106. 24212578 PMC3911106

[56] Fischer M, Rulicke T, Raeber A, Sailer A, Moser M, Oesch B, et al Prion protein (PrP) with amino-proximal deletions restoring susceptibility of PrP knockout mice to scrapie. EMBO J. 1996;15(6):1255–64. PubMed Central PMCID: PMCPMC450028. 8635458 PMC450028

[57] Green R, Horrocks C, Wilkinson A, Hawkins SA, Ryder SJ. Primary isolation of the bovine spongiform encephalopathy agent in mice: agent definition based on a review of 150 transmissions. J Comp Pathol. 2005;132(2–3):117–31. Epub 2005/03/02. 10.1016/j.jcpa.2004.08.002 . 15737338

[58] Espinosa JC, Andreoletti O, Castilla J, Herva ME, Morales M, Alamillo E, et al Sheep-passaged bovine spongiform encephalopathy agent exhibits altered pathobiological properties in bovine-PrP transgenic mice. J Virol. 2007;81(2):835–43. Epub 2006/11/03. 10.1128/JVI.01356-06 PubMed PMID: 17079295; PubMed Central PMCID: PMCPmc1797487. 17079295 PMC1797487

[59] Priem J, Langeveld JPM, van Keulen LJM, van Zijderveld FG, Andreoletti O, Bossers A. Enhanced Virulence of Sheep-Passaged Bovine Spongiform Encephalopathy Agent Is Revealed by Decreased Polymorphism Barriers in Prion Protein Conversion Studies. Journal of Virology. 2014;88(5):2903–12. 10.1128/JVI.02446-13 PubMed PMID: PMC3958111. 24371051 PMC3958111

[60] Moreno JA, Telling GC. Insights into Mechanisms of Transmission and Pathogenesis from Transgenic Mouse Models of Prion Diseases. Methods Mol Biol. 2017;1658:219–52. 10.1007/978-1-4939-7244-9_16 PubMed Central PMCID: PMCPMC5902812. 28861793 PMC5902812

[61] Watts JC, Prusiner SB. Mouse models for studying the formation and propagation of prions. J Biol Chem. 2014;289(29):19841–9. 10.1074/jbc.R114.550707 ; PubMed Central PMCID: PMCPMC4106304. 24860095 PMC4106304

[62] Chen SG, Teplow DB, Parchi P, Teller JK, Gambetti P, Autilio-Gambetti L. Truncated forms of the human prion protein in normal brain and in prion diseases. J Biol Chem. 1995;270(32):19173–80. Epub 1995/08/11. . 7642585 10.1074/jbc.270.32.19173

[63] Langeveld JP, Erkens JH, Rammel I, Jacobs JG, Davidse A, van Zijderveld FG, et al Four independent molecular prion protein parameters for discriminating new cases of C, L, and h bovine spongiform encephalopathy in cattle. J Clin Microbiol. 2011;49(8):3026–8. 10.1128/JCM.01102-11 ; PubMed Central PMCID: PMCPMC3147744. 21677067 PMC3147744

[64] Croset A, Delafosse L, Gaudry JP, Arod C, Glez L, Losberger C, et al Differences in the glycosylation of recombinant proteins expressed in HEK and CHO cells. J Biotechnol. 2012;161(3):336–48. Epub 2012/07/21. 10.1016/j.jbiotec.2012.06.038 . 22814405

[65] DeArmond SJ, Qiu Y, Sanchez H, Spilman PR, Ninchak-Casey A, Alonso D, et al PrPc glycoform heterogeneity as a function of brain region: implications for selective targeting of neurons by prion strains. J Neuropathol Exp Neurol. 1999;58(9):1000–9. Epub 1999/09/28. . 10499442 10.1097/00005072-199909000-00010

[66] Pan T, Colucci M, Wong BS, Li RL, Liu T, Petersen RB, et al Novel differences between two human prion strains revealed by two-dimensional gel electrophoresis. Journal of Biological Chemistry. 2001;276(40):37284–8. 10.1074/jbc.M107358200 11489910

[67] Pan T, Li RR, Wong BS, Liu T, Gambetti P, Sy MS. Heterogeneity of normal prion protein in two-dimensional immunoblot: presence of various glycosylated and truncated forms. Journal of Neurochemistry. 2002;81(5):1092–101. 12065622 10.1046/j.1471-4159.2002.00909.x

[68] Pirisinu L, Di Bari M, Marcon S, Vaccari G, D'Agostino C, Fazzi P, et al A new method for the characterization of strain-specific conformational stability of protease-sensitive and protease-resistant PrPSc. PLoS One. 2010;5(9):e12723 Epub 2010/09/22. 10.1371/journal.pone.0012723 ; PubMed Central PMCID: PMCPmc2939050. 20856860 PMC2939050

