# Supplementary material for: Molecular characterisation of atypical BSE prions by mass spectrometry and changes following transmission to sheep and transgenic mouse models
Source: PLoS One. 2018 Nov 8;13(11):e0206505. doi: 10.1371/journal.pone.0206505 (PMC6224059; doi:10.1371/journal.pone.0206505)
Supplement: S1 Fig — Animal ID/Sample references as in Table 1. Lanes for samples not relevant to the present work have been edited out. Where blots for sample used in MS studies were not available, a WB from a littermate was included, indicated by an additional: M7L: littermate of M7, M11L: littermate of M11, M18L: littermate of M18. Scr = ovine classical scrapie positive control BSE = bovine classical BSE positive control. All blots were run using 12% BisTris gels and Magic Mark XP molecular mass markers (Thermo Fisher) were used. (PDF) [file pone.0206505.s001.pdf]

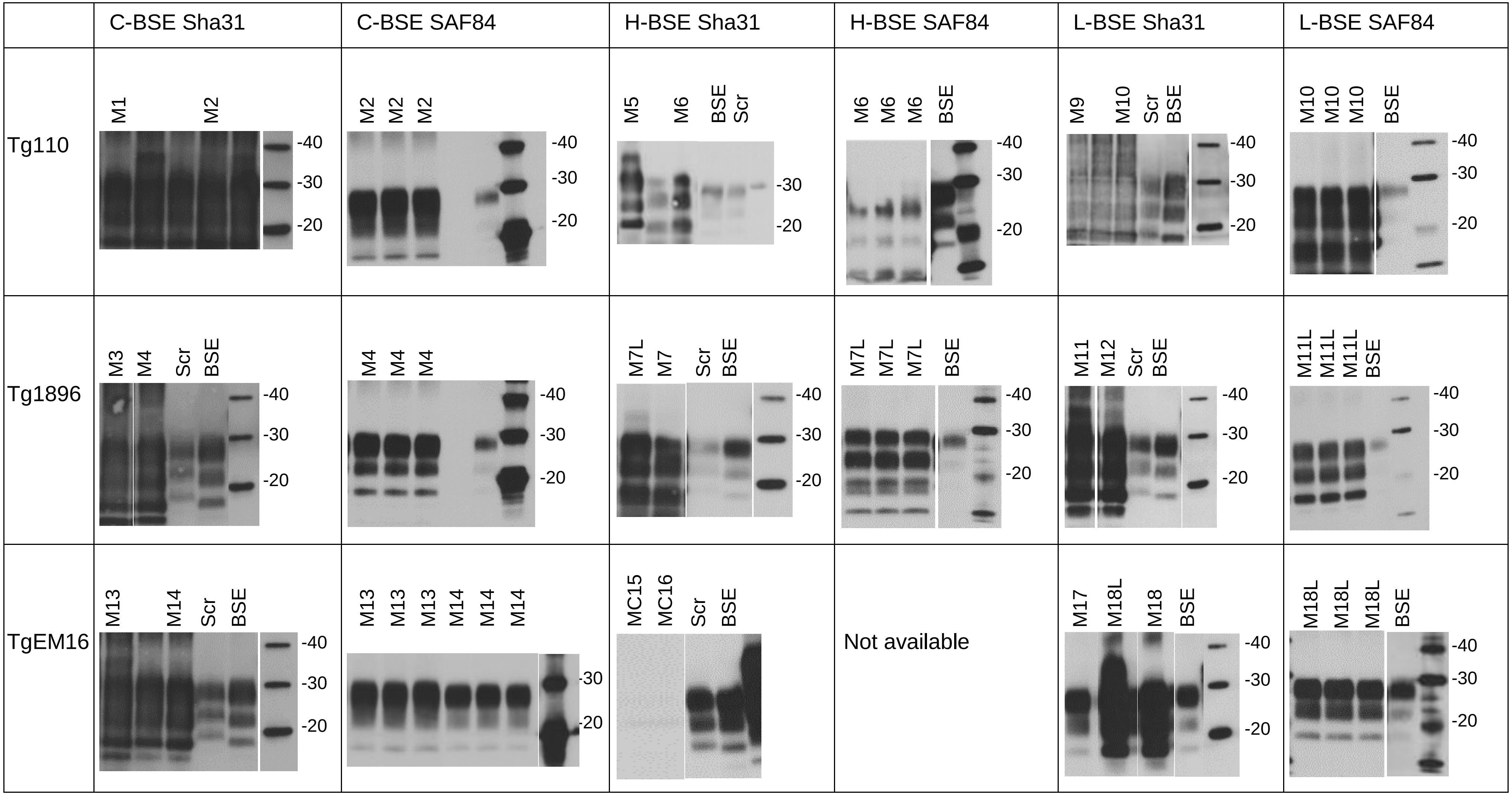

S1 Fig. Western blots of C-, H- and L-BSE transmitted to transgenic mouse lines Tg110, Tg1896 and TgEM16. Animal ID/Sample references as in Table 1. Lanes for samples not relevant to the present work have been edited out. Where blots for sample used in MS studies were not available, a WB from a littermate was included, indicated by an additional: M7L: littermate of M7, M11L: littermate of M11, M18L: littermate of M18. Scr=ovine classical scrapie positive control BSE = bovine classical BSE positive control. All blots were run using 12% BisTris gels and Magic Mark XP molecular mass markers (Thermo Fisher) were used.
