# Supplementary material for: Molecular characterisation of atypical BSE prions by mass spectrometry and changes following transmission to sheep and transgenic mouse models
Source: PLoS One. 2018 Nov 8;13(11):e0206505. doi: 10.1371/journal.pone.0206505 (PMC6224059; doi:10.1371/journal.pone.0206505)
Supplement: S2 Fig — Arrowheads indicate trypsin cleavage sites. Amino acid residues in bold indicate interspecies polymorphisms in comparison with the bovine sequence. For polymorphisms R154H or Q171R, tryptic peptide numbering is maintained. (PDF) [file pone.0206505.s002.pdf]

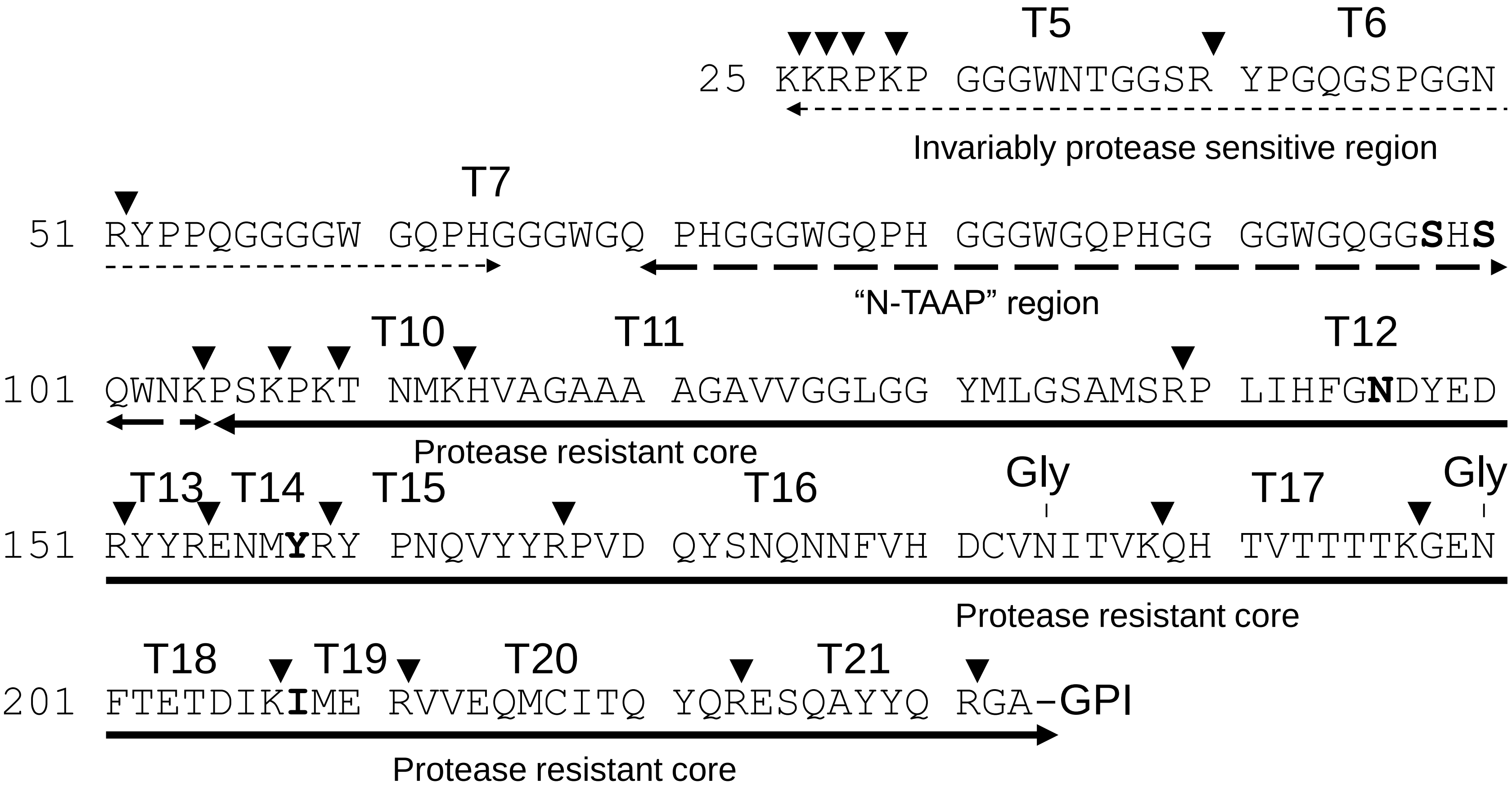


**S2 Fig.** Ovine PrP protein sequence (136A/154R/171Q) indicating N-TAAP region and tryptic peptides used in the mass spectrometry-based assay (also refer to S3 and S4 Table). Arrowheads indicate trypsin cleavage sites. Amino acid residues in bold indicate interspecies polymorphisms in comparison with the bovine sequence. For polymorphisms R154H or Q171R, tryptic peptide numbering is maintained.
