# Supplementary material for: Molecular characterisation of atypical BSE prions by mass spectrometry and changes following transmission to sheep and transgenic mouse models
Source: PLoS One. 2018 Nov 8;13(11):e0206505. doi: 10.1371/journal.pone.0206505 (PMC6224059; doi:10.1371/journal.pone.0206505)
Supplement: S3 Fig — Case references: (A,D) 06/06990, (B,E) 1643/97, (C,F) 1339/96. Samples (A-C) were prepared using 350 mg, (D-F) using 1000 mg starting material, divided in four prior to PK treatment, and processed and analysed in parallel, giving TEmax of 3.5 and 7 mg, resp., then data were combined to create the profiles. Where error bars exceeded the maximum of the y-axis range displayed, they were clipped by the software and drawn in manually. Peptide numbering is as detailed in Fig 1, and in S1 and S2 Tables. (PDF) [file pone.0206505.s003.pdf]

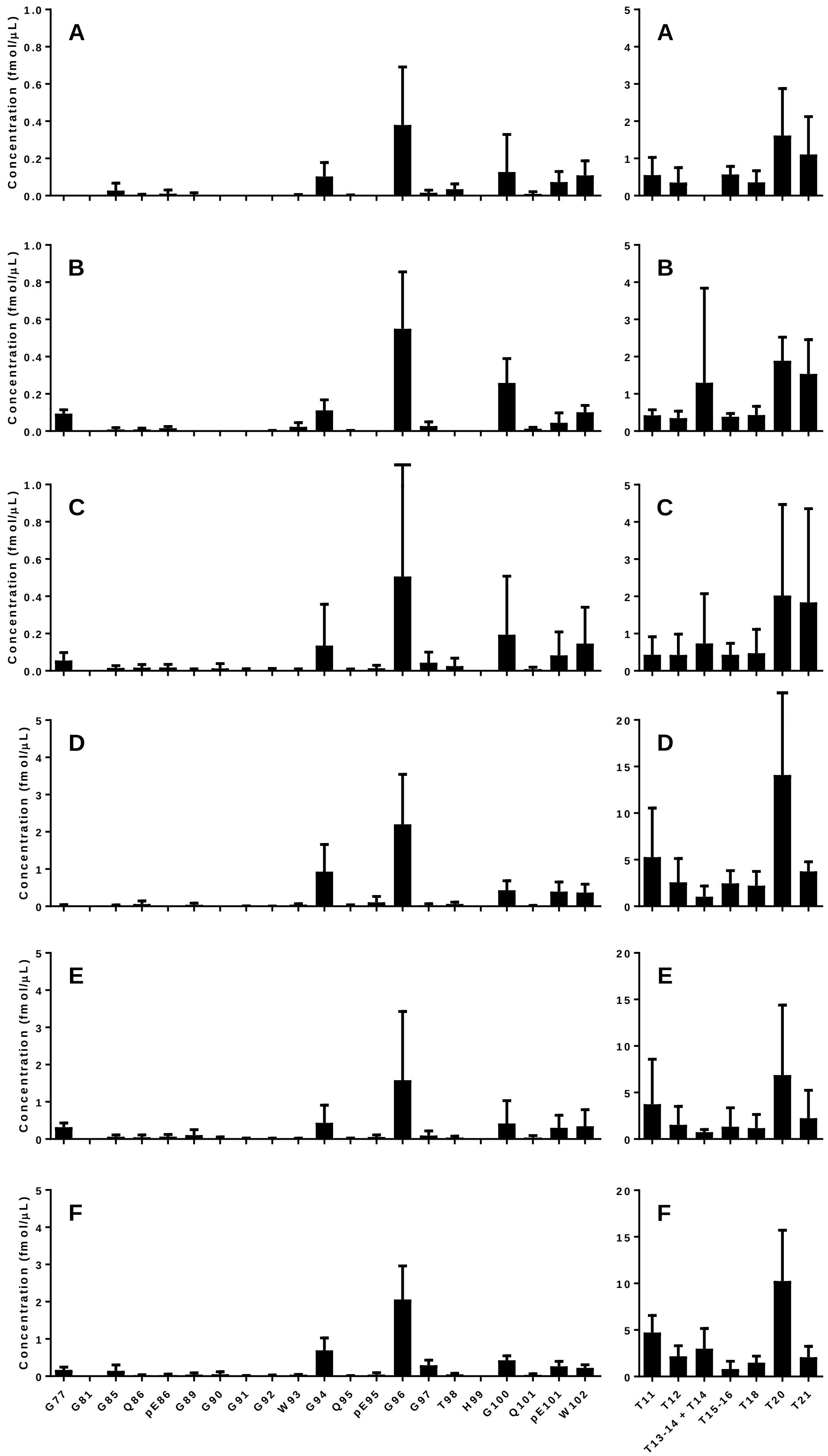
A
B
C
D
E
F
Concentration (fmol/µL)
G77
G81
G85
Q86
pE86
G89
G90
G91
G92
W93
G94
Q95
pE95
G96
G97
T98
H99
G100
Q101
pE101
W102
T11
T12
T13-14 + T14
T15-16
T18
T20
T21

S3 Fig. Absolute N-TAAP (left-hand panels) and tryptic peptide profiles (right hand panels) of natural b**ovine classical BSE cases from the UK.** Case references: (A,D) 06/06990, (B,E) 1643/97, (C,F) 1339/96. Samples (A-C) were prepared using 350 mg, (D-F) using 1000 mg starting material, divided in four prior to PK treatment, and processed and analysed in parallel, giving TE_{max} of 3.5 and 7 mg, resp., then data were combined to create the profiles. Where error bars exceeded the maximum of the y-axis range displayed, they were clipped by the software and drawn in manually. Peptide numbering is as detailed in Fig 1, and in S3 and S4 Tables.
