# Supplementary material for: Molecular characterisation of atypical BSE prions by mass spectrometry and changes following transmission to sheep and transgenic mouse models
Source: PLoS One. 2018 Nov 8;13(11):e0206505. doi: 10.1371/journal.pone.0206505 (PMC6224059; doi:10.1371/journal.pone.0206505)
Supplement: S4 Fig — Absolute abundances N-TAAP (left-hand panels) and tryptic peptide profiles (right hand panels). H3, H4: H-BSE primary passage cases [37] L2, L4: L-BSE primary passage cases [37]; L6: L-BSE secondary passage case [38]. Samples (350 mg) were divided into two replicates prior to PK treatment and processed and analysed in parallel (TEmax = 7 mg), then data combined to create the profiles. Where error bars exceeded the maximum of the y-axis range displayed, they were clipped by the software and drawn in manually. (PDF) [file pone.0206505.s004.pdf]

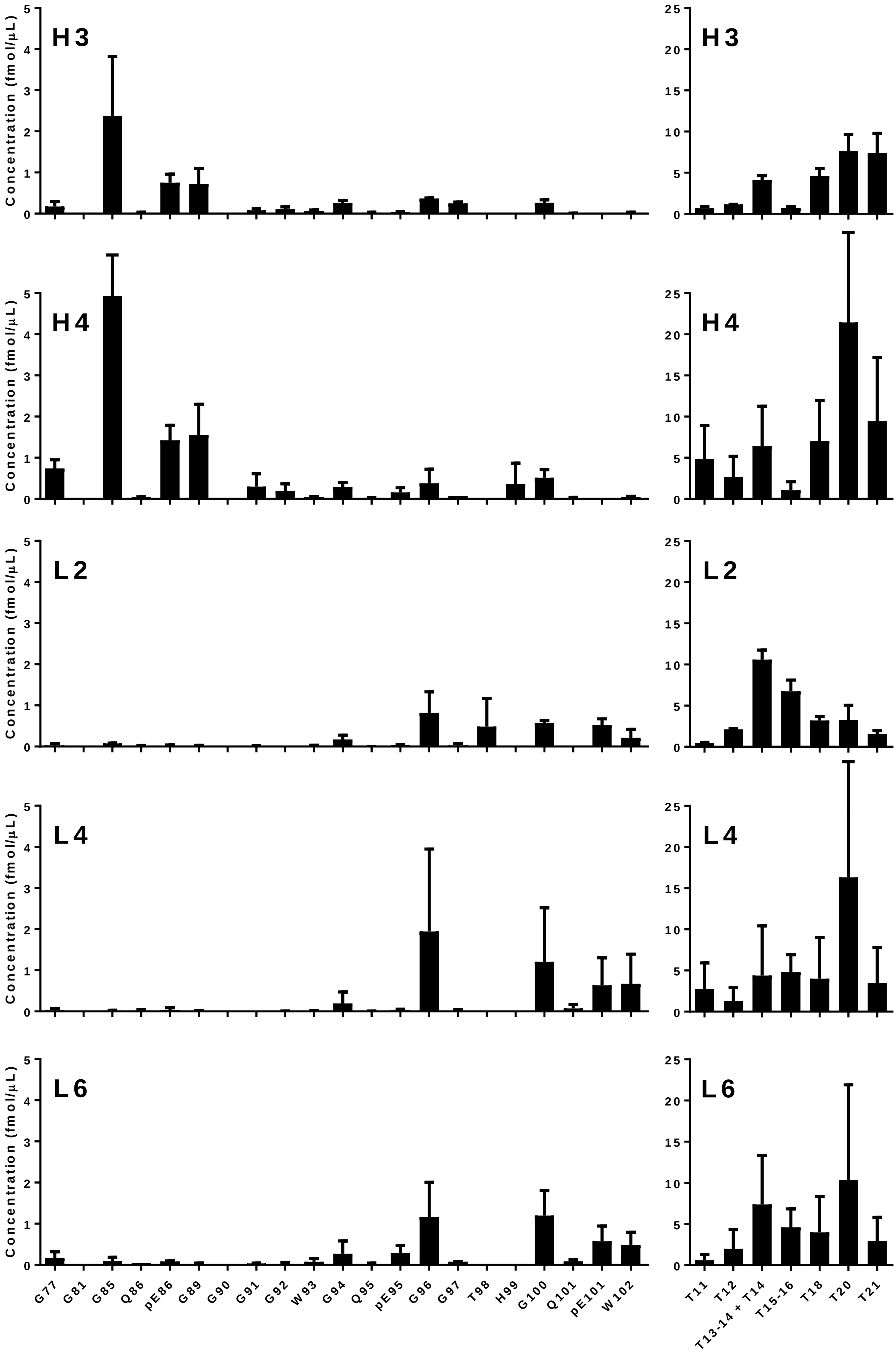
H3
H4
L2
L4
L6
Concentration (fmol/µL)
G77
G81
G85
Q86
pE86
G89
G90
G91
G92
W93
G94
Q95
pE95
G96
G97
T98
H99
G100
Q101
pE101
W102
T11
T12
T13-14 + T14
T15-16
T18
T20
T21

S4 Fig. Bovine atypical BSE profiles. Absolute abundances N-TAAP (left-hand panels) and tryptic peptide profiles (right hand panels). H3, H4: H-BSE primary passage cases [37] L2, L4: L-BSE primary passage cases [37]; L6: L-BSE secondary passage case [38]. Samples (350 mg) were divided into two replicates prior to PK treatment and processed and analysed in parallel (TE_{max} = 7 mg), then data combined to create the profiles. Where error bars exceeded the maximum of the y-axis range displayed, they were clipped by the software and drawn in manually.
