# Supplementary material for: Molecular characterisation of atypical BSE prions by mass spectrometry and changes following transmission to sheep and transgenic mouse models
Source: PLoS One. 2018 Nov 8;13(11):e0206505. doi: 10.1371/journal.pone.0206505 (PMC6224059; doi:10.1371/journal.pone.0206505)
Supplement: S5 Fig — N-TAAP (left-hand panels) and tryptic peptide profiles (right hand panels) from transgenic mice inoculated with C-BSE from a single UK source. Samples (200–300 mg) were divided into two replicates prior to PK treatment and processed and analysed in parallel, then data were combined to create the profiles. M1, M2: Tg110; M3, M4: Tg1896. TEmax M1 = 5.5, M2 = 4.2, M3 = 4.5, M4 = 5.8. (PDF) [file pone.0206505.s005.pdf]

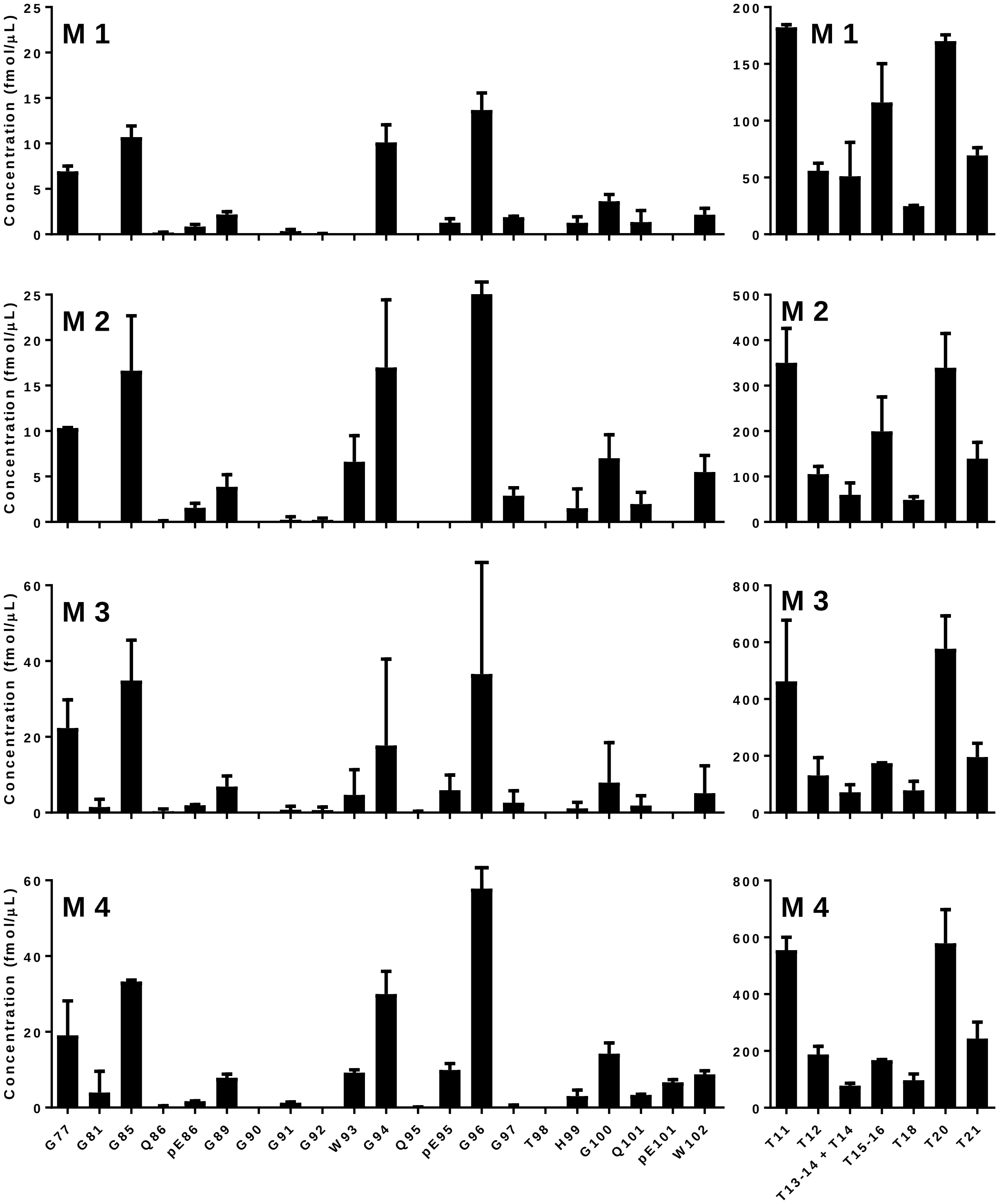

S5 Fig. C-BSE into bovinised mice. N-TAAP (left-hand panels) and tryptic peptide profiles (right hand panels) from transgenic mice inoculated with C-BSE from a single UK source. Samples (200-300 mg) were divided into two replicates prior to PK treatment and processed and analysed in parallel, then data were combined to create the profiles. M1, M2: Tg110; M3, M4: Tg1896. TE_{max} M1 = 5.5, M2=4.2, M3=4.5, M4=5.8.
