# Supplementary material for: Molecular characterisation of atypical BSE prions by mass spectrometry and changes following transmission to sheep and transgenic mouse models
Source: PLoS One. 2018 Nov 8;13(11):e0206505. doi: 10.1371/journal.pone.0206505 (PMC6224059; doi:10.1371/journal.pone.0206505)
Supplement: S7 Fig — Absolute abundance N-TAAP (left-hand panels) and tryptic peptide profiles (right hand panels) from transgenic mice inoculated with L-BSE from a single UK source. M9, M10: Tg110; M11, M12: Tg1896. Samples (approx. 100mg for Tg110 and 200mg for Tg1896) were divided into two replicates prior to PK treatment and processed and analysed in parallel, then data were combined to create the profiles. TEmax: M9 = 1.8; M10 = 1.8, M11 = 3.4, M12 = 3.8. (PDF) [file pone.0206505.s007.pdf]

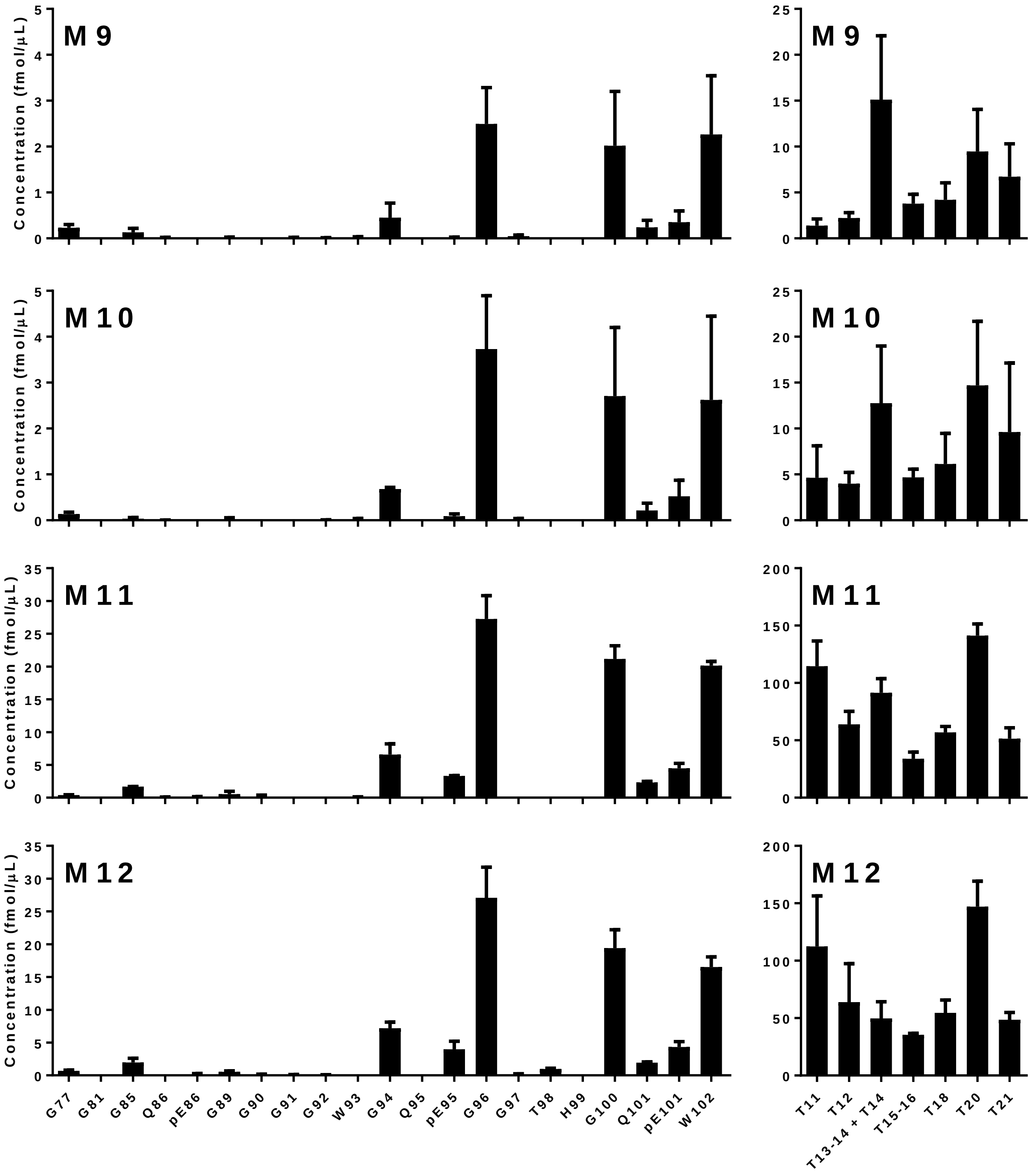

S7 Fig. L-BSE profiles obtained from individual bovinised mice. N-TAAP (left-hand panels) and tryptic peptide profiles (right hand panels) from transgenic mice inoculated with L-BSE from a single UK source. M9, M10: Tg110; M11, M12: Tg1896. Samples (approx. 100mg for Tg110 and 200mg for Tg1896) were divided into two replicates prior to PK treatment and processed and analysed in parallel, then data were combined to create the profiles. TE_{max}: M9=1.8; M10=1.8, M11=3.4, M12=3.8.
