# Supplementary material for: Molecular characterisation of atypical BSE prions by mass spectrometry and changes following transmission to sheep and transgenic mouse models
Source: PLoS One. 2018 Nov 8;13(11):e0206505. doi: 10.1371/journal.pone.0206505 (PMC6224059; doi:10.1371/journal.pone.0206505)
Supplement: S8 Fig — Absolute abundance N-TAAP (left-hand side) and tryptic peptide profiles (right hand side) from sheep inoculated with the same L-BSE source as used in the bovine experimental transmissions, as described in [19]. (A) animal ID 456/11 ALRQ/VRQ, (B) 140/11, ALRQ/VRQ (C) 267/11 VRQ/VRQ (Dorset males) and (D) 457/11 AFRQ/AFRQ (Cheviot). T11+ denotes the summed contributions of T11, T11-12 and T11-13. T12 L141 denotes the T12 variant containing the L141 allele; T12 F141 denotes the T12 variant containing the F141 allele. Samples (approx. 350 mg) were divided into two replicates prior to PK treatment and processed and analysed in parallel, then data combined to create the profiles. TEmax = 7mg for each profile. (PDF) [file pone.0206505.s008.pdf]

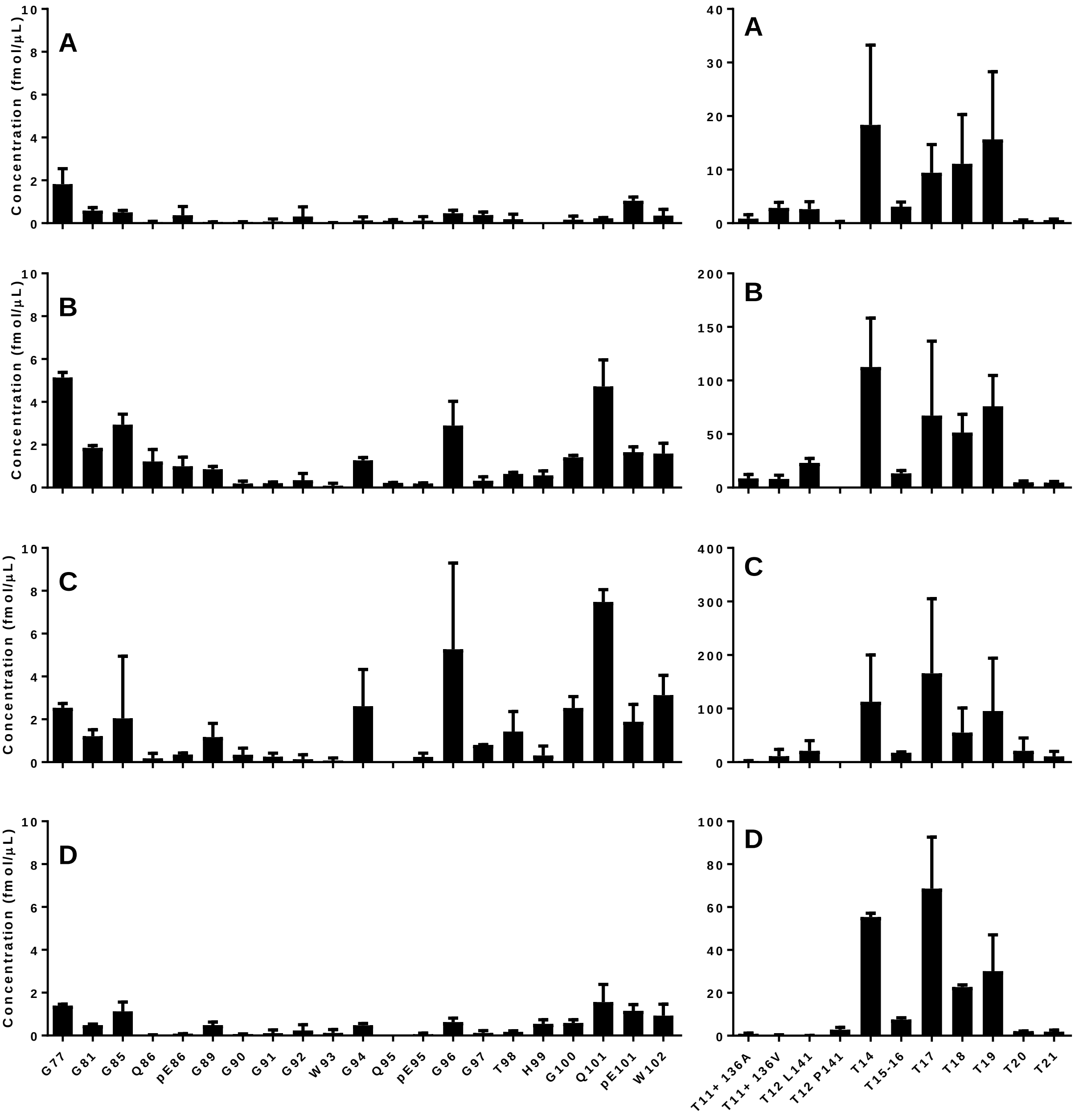

S8 Fig. L-BSE into sheep. N-TAAP (left-hand side) and tryptic peptide profiles (right hand side) from sheep inoculated with the same L-BSE source as used in the bovine experimental transmissions, as described in [18]. (A) animal ID 456/11 ALRQ/VRQ, (B) 140/11, ALRQ/VRQ (C) 267/11 VRQ/VRQ (Dorset males) and (D) 457/11 AFRQ/AFRQ (Cheviot). T11+ denotes the summed contributions of T11, T11-12 and T11-13. Samples (approx. 350 mg) were divided into two replicates prior to PK treatment and processed and analysed in parallel, then data combined to create the profiles. TE_{max}= 7mg for each profile.
