# Supplementary material for: Molecular characterisation of atypical BSE prions by mass spectrometry and changes following transmission to sheep and transgenic mouse models
Source: PLoS One. 2018 Nov 8;13(11):e0206505. doi: 10.1371/journal.pone.0206505 (PMC6224059; doi:10.1371/journal.pone.0206505)

S9 Fig. (A, B) N-TAAP and tryptic peptide chromatograms of C-BSE in a TgEM16 mouse (M13). The y-axes have been scaled to the compound with the highest abundance of the set. This is determined by concentration and detection response.

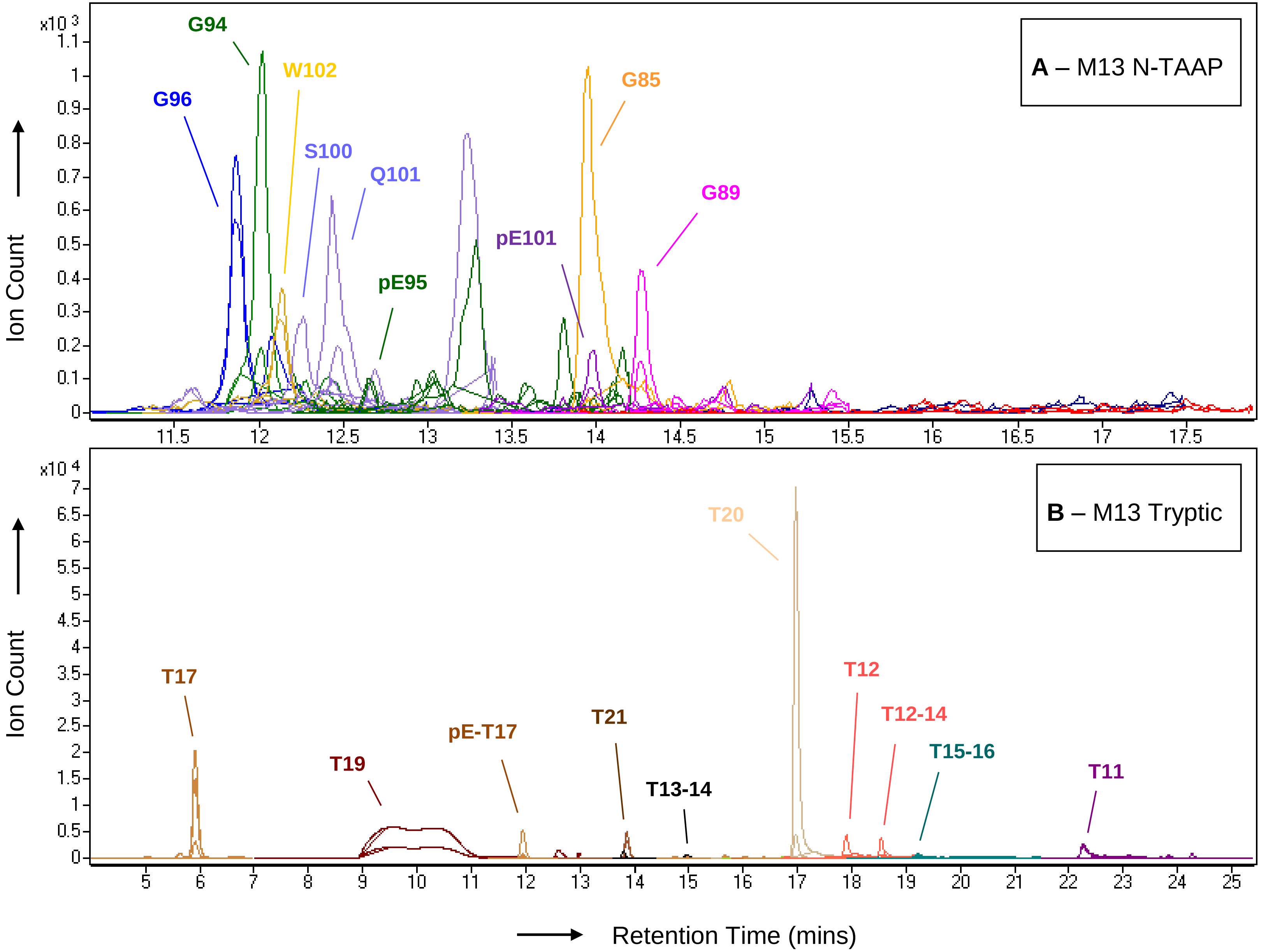

S9 Fig. (C, D) N-TAAP and tryptic peptide chromatograms of H-BSE in a TgEM16 mouse (M15). The y-axes have been scaled to the compound with the highest abundance of the set. This is determined by concentration and detection response.

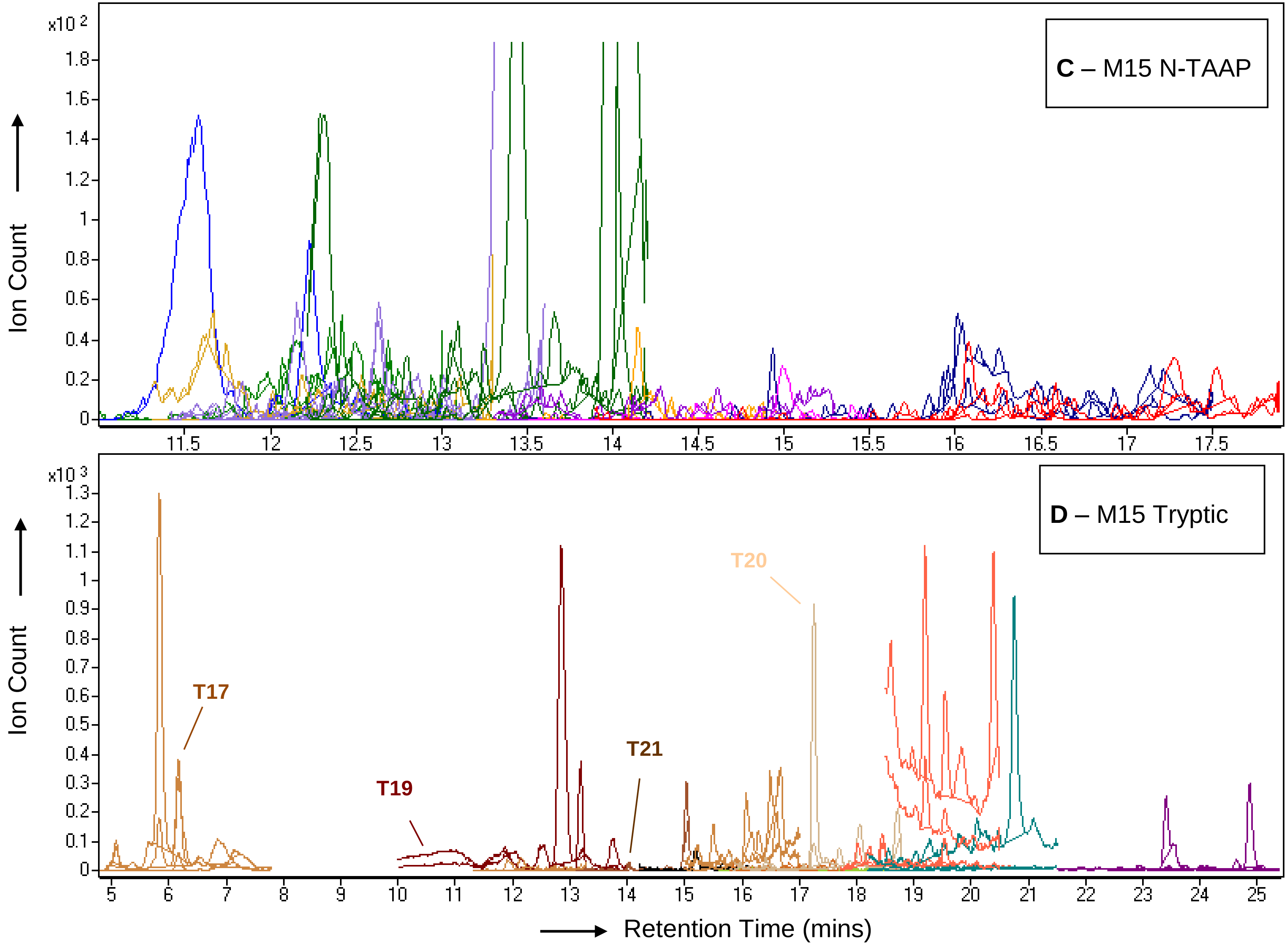

S9 Fig. (E, F) N-TAAP and tryptic peptide chromatograms of L-BSE in a TgEM16 mouse (M17). The y-axes have been scaled to the compound with the highest abundance of the set. This is determined by concentration and detection response.

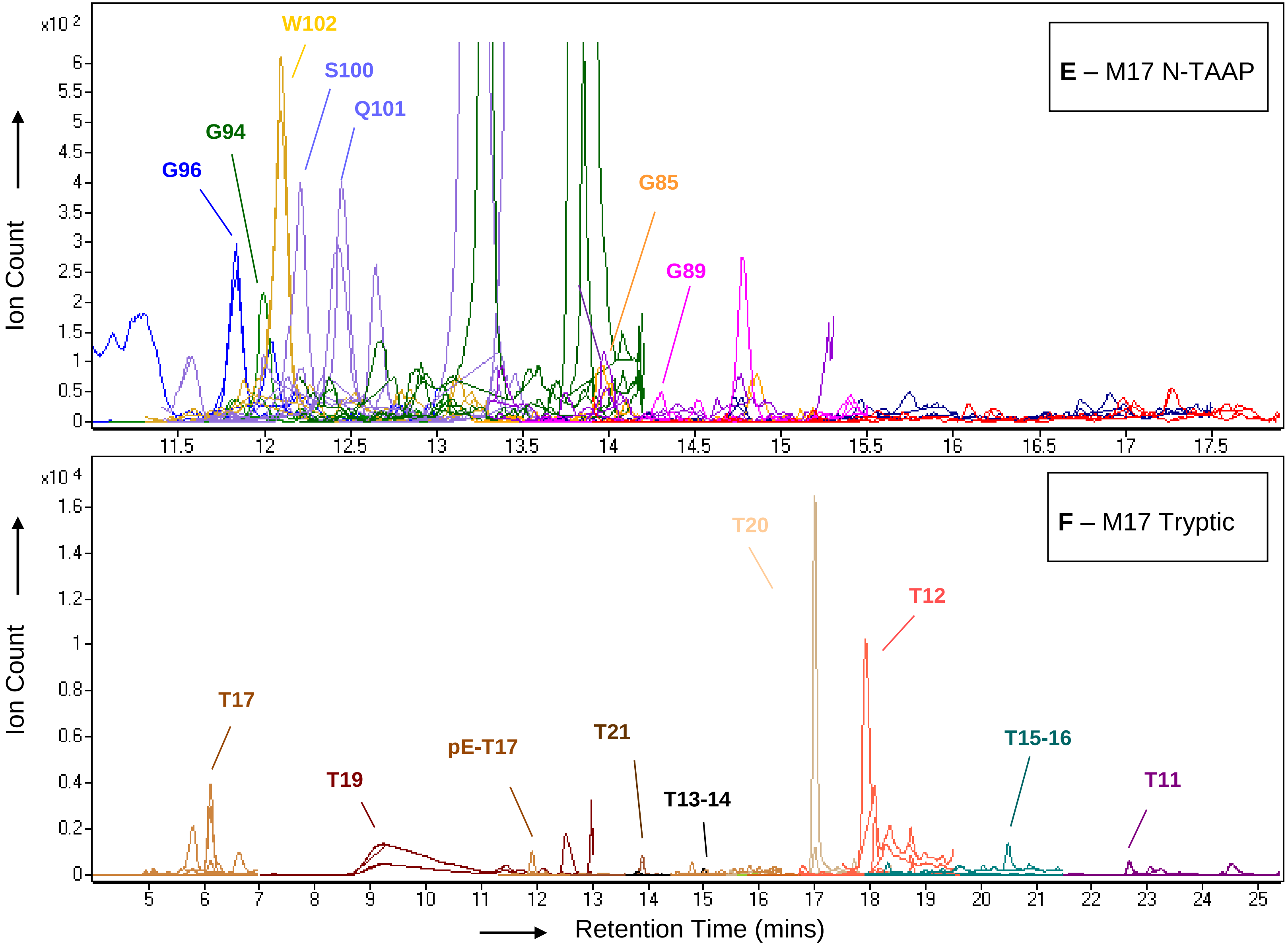

S9 Fig. (G, H) N-TAAP and tryptic peptide chromatograms of L-BSE in an ARQ/VRQ sheep (267/11). The y-axes have been scaled to the compound with the highest abundance of the set. This is determined by concentration and detection response.

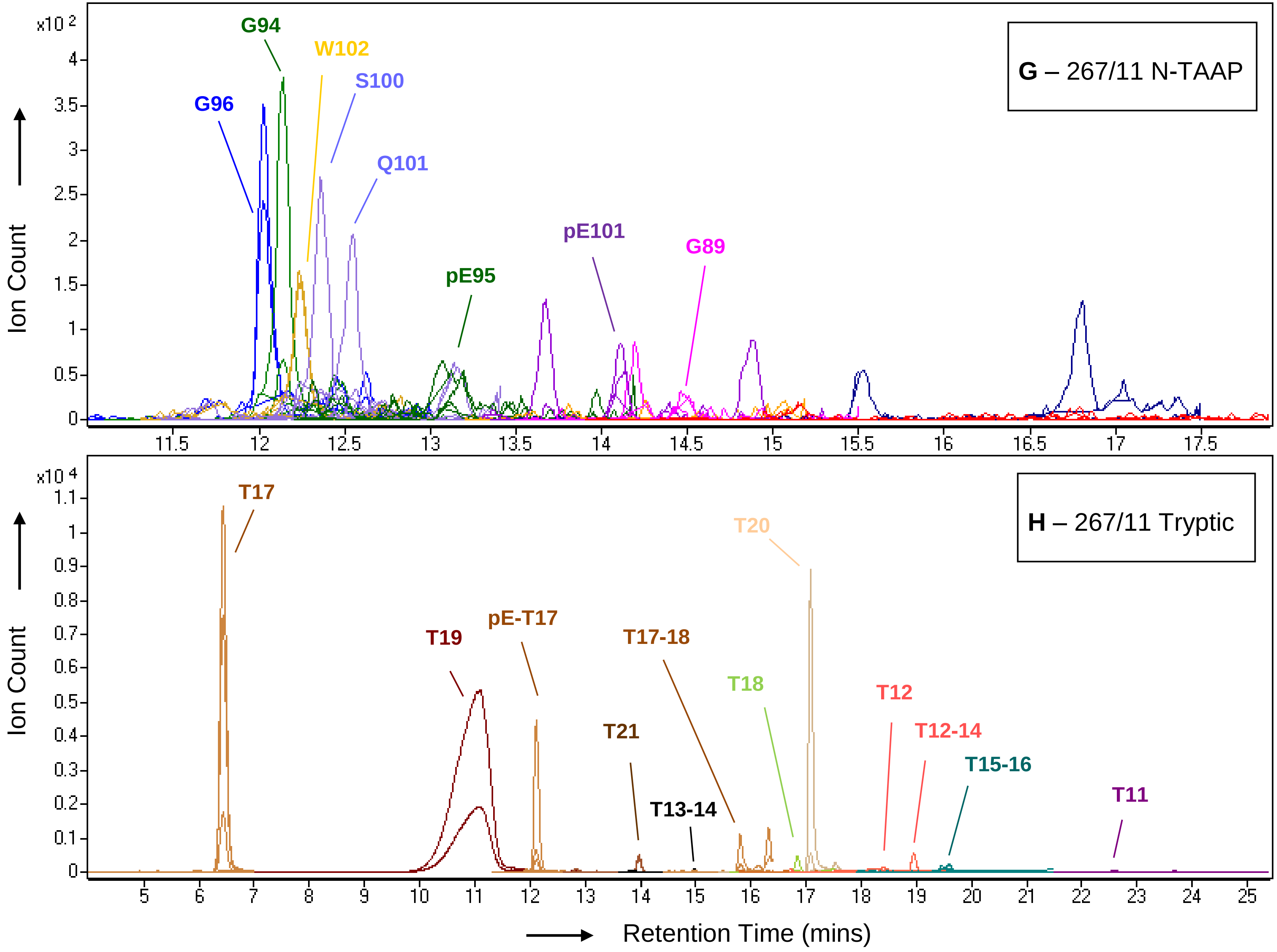

S9 Fig. (I, J) N-TAAP and tryptic peptide chromatograms of C-BSE in an AHQ/AHQ sheep (822/05). The y-axes have been scaled to the compound with the highest abundance of the set. This is determined by concentration and detection response.

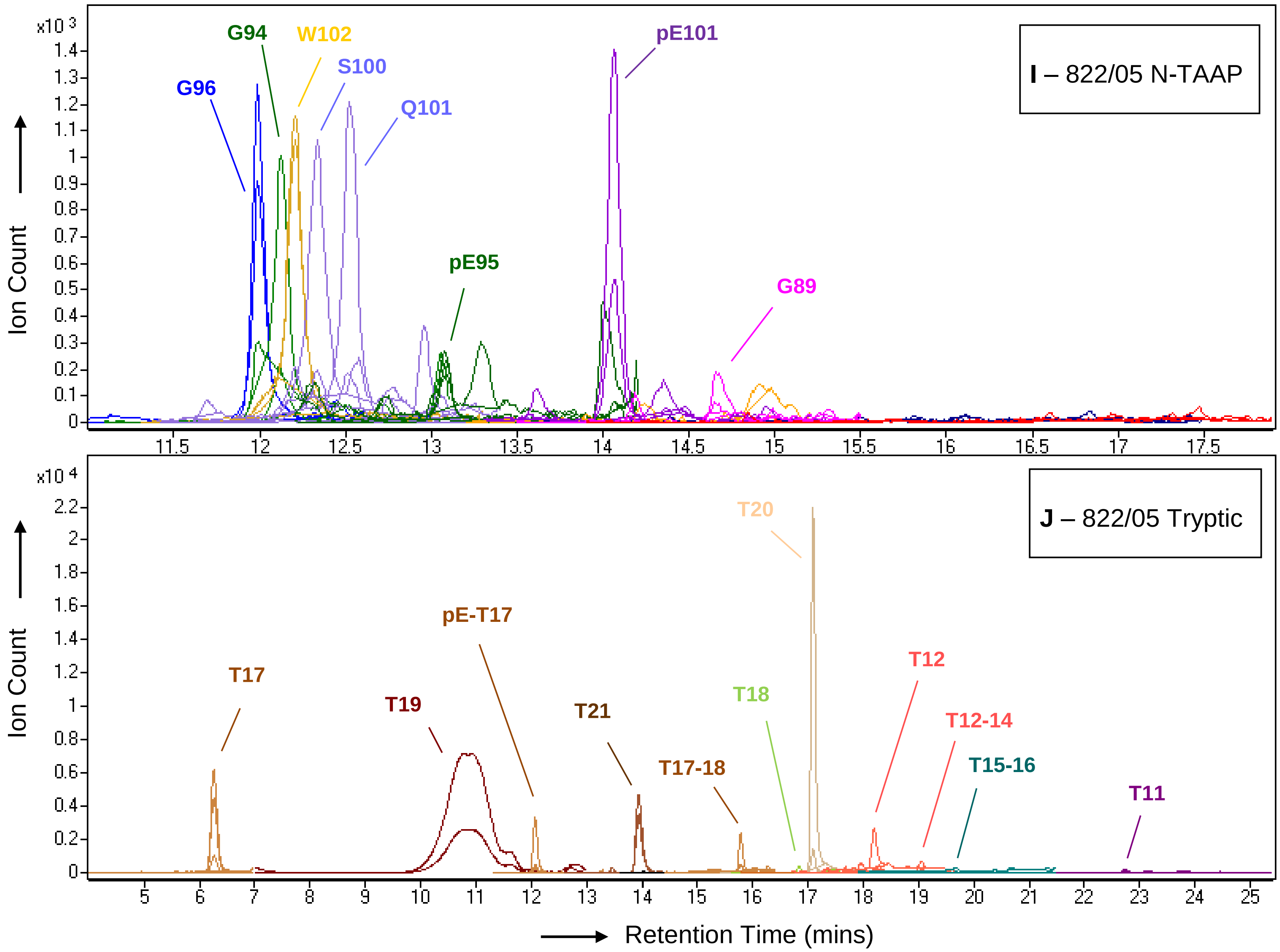

S9 Fig. (K, L) N-TAAP and tryptic peptide chromatograms from CH1641 scrapie in an AHQ/AHQ sheep (851/05). The y-axes have been scaled to the compound with the highest abundance of the set. This is determined by concentration and detection response.

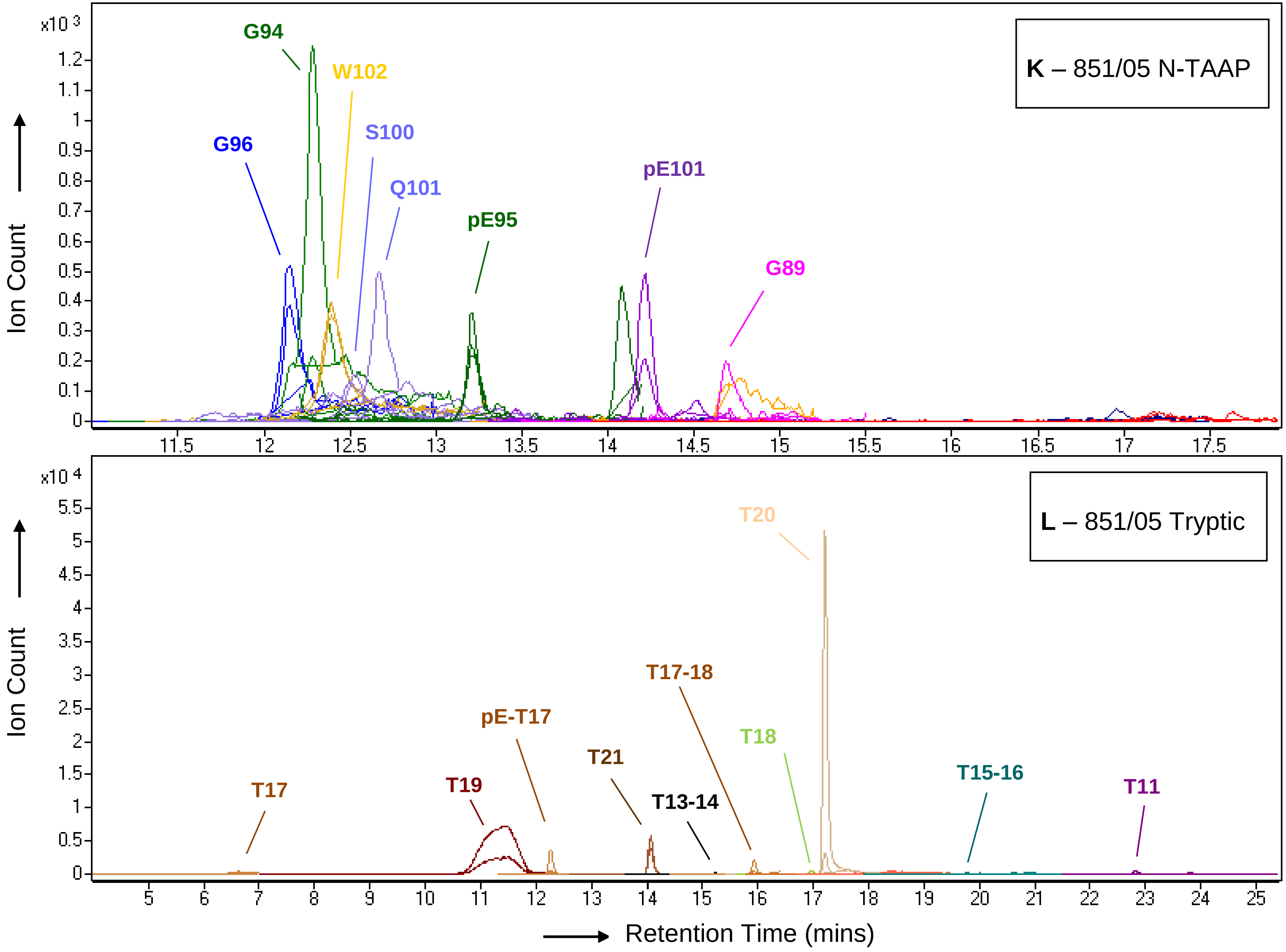

Supplement: S9 Fig — (A, B) N-TAAP and tryptic peptide chromatograms of C-BSE in a TgEM16 mouse (M13). (C, D) N-TAAP and tryptic peptide chromatograms of H-BSE in a TgEM16 mouse (M15). (E, F) N-TAAP and tryptic peptide chromatograms of L-BSE in a TgEM16 mouse (M17). (G, H) N-TAAP and tryptic peptide chromatograms of L-BSE in an ARQ/VRQ sheep (267/11). (I, J) N-TAAP and tryptic peptide chromatograms of C-BSE in an AHQ/AHQ sheep (822/05). (K, L) N-TAAP and tryptic peptide chromatograms from CH1641 scrapie in an AHQ/AHQ sheep (851/05). (PDF) [file pone.0206505.s009.pdf]
