# Supplementary material for: Molecular characterisation of atypical BSE prions by mass spectrometry and changes following transmission to sheep and transgenic mouse models
Source: PLoS One. 2018 Nov 8;13(11):e0206505. doi: 10.1371/journal.pone.0206505 (PMC6224059; doi:10.1371/journal.pone.0206505)
Supplement: S1 Table — Amino acid residues in bold: bovine/ovine interspecies polymorphisms; underlined: ovine intraspecies polymorphisms. pE denotes a pyroglutamyl N-terminal amino acid residue. Cep denotes the ethylpyridyl alkylated product of cysteine; N* indicates an N-glycosylation site. (PDF) [file pone.0206505.s010.pdf]

S1 Table. Bovine and ovine PrP tryptic peptides and how these have been included in the assays and in plots. Amino acid residues in **bold**: bovine/ovine interspecies polymorphisms; underlined: ovine intraspecies polymorphisms. pE denotes a pyroglutamyl N-terminal amino acid residue. C^{ep} denotes the ethylpyridyl alkylated product of cysteine; N* indicates an N-glycosylation site.

Tryptic peptide	Bovine	In Assay	Plotted	Ovine	In Assay	Plotted
T1	25K	×	×	25K	×	×
T2	26K	×	×	26K	×	×
T3	27R	×	×	27R	×	×
T4	28PK29	×	×	28PK29	×	×
T5	30PGGGWNTGGSR40	×	×	30PGGGWNTGGSR40	×	×
T6	41YPGQGSPGGNR51[a]	✓	×	41YPGQGSPGGNR51[a]	✓	×
T7	52YPPQGGGGWGQPHGGGWGQPHGGGWGQPHGGGWGQPH GGGGWGQGG**T**H**G**QWNK104	×	×	52YPPQGGGGWGQPHGGGWGQPHGGGWGQPHGGGWGQPH GGGGWGQGG**S**H**S**QWNK104	×	×
T8	105PSK107	×	×	105PSK107	×	×
T9	108PK109	×	×	108PK109	×	×
T10	110TNMK113[b]	×	×	110TNMK113[b]	×	×
T11	114HVAGAAAAGAVVGGLGGYMLGSAMSR139	✓	✓	114HVAGAAAAGAVVGGLGGYMLGSAMSR139<br>114HVAGAAAAGAVVGGLGGYMLGSVMSR139	✓<br>✓	✓<br>✓
T12	140PLIHFG**S**DYEDR151	✓	✓	140PLIHFG**N**DYEDR151<br>140PFIHFG**N**DYEDR151	✓<br>✓	✓<br>✓
T13	152YYR154[b]	×	×	152YYR154[b]	×	×
T14	155ENM**H**R159[b]	✓	✓[c]	155ENM**Y**R159	✓	✓
T15	160YPNQVYYR167	✓	×[d]	160YPNQVYYR167	✓	×[d]
T16	168PVDQYSNQNNFVHDC^{ep}VN*ITVK188	✓	×[d]	168PVDQYSNQNNFVHDC^{ep}VN*ITVK188<br>168PVDHYSNQNNFVHDC^{ep}VN*ITVK188<br>168PVDR171	✓<br>✓<br>✓	×[d]<br>×<br>×
T16'	n/a			172YSNQNNFVHDC^{ep}VN*ITVK188	✓	×
T17	189 **E**HTVTTTTK197[b]	×	×	189 **Q**HTVTTTTK197<br>189pEHTVTTTTK197	✓<br>✓	✓<br>×

T18	198GEN*FTETDIK207	✓	✓	198GEN*FTETDIK207	✓	✓
T19	208**M**MER211[b]	×	×	208**I**MER211	✓	✓
T20	212VVEQMC^{ep}ITQYQR223	✓	✓	212VVEQMC^{ep}ITQYQR223	✓	✓
T21	224ESQAYYQR231	✓	✓	224ESQAYYQR231	✓	✓
T11-T12	114HVAGAAAAGAVVGGLGGYMLGSAMSRPLIHFG**S**DYEDR151	×	×	114HVAGAAAAGAVVGGLGGYMLGSAMSRPLIHFG**N**DYEDR151<br>114HVAGAAAAGAVVGGLGGYMLGSVMSRPLIHFG**N**DYEDR151<br>114HVAGAAAAGAVVGGLGGYMLGSAMSRPFIHFG**N**DYEDR151	✓<br>✓<br>×	✓<br>✓<br>×
T11-T13	114HVAGAAAAGAVVGGLGGYMLGSAMSRPLIHFG**S**DYEDRYYR154	×	×	114HVAGAAAAGAVVGGLGGYMLGSAMSRPLIHFG**N**DYEDRYYR154<br>114HVAGAAAAGAVVGGLGGYMLGSVMSRPLIHFG**N**DYEDRYYR154<br>114HVAGAAAAGAVVGGLGGYMLGSAMSRPFIHFG**N**DYEDRYYR154	✓<br>✓<br>×	×<br>×<br>×
T13-T14	152YYRENM**H**R159	✓	✓[c]	152YYRENM**Y**R159<br>152YYHENM**Y**R159	✓<br>✓	×<br>×/✓[e]
T15-T16	160YPNQVYYRPVDQYSNQNNFVHDC^{ep}VN*ITVK188	✓	✓	160YPNQVYYRPVDQYSNQNNFVHDC^{ep}VN*ITVK188<br>160YPNQVYYRPVDHYSNQNNFVHDC^{ep}VN*ITVK188<br>160YPNQVYYRPVDR171	✓<br>✓<br>✓	✓<br>×<br>×
T17-T18	189 **E**HTVTTTTKGEN*FTETDIK207	×	×	189 **Q**HTVTTTTKGEN*FTETDIK207<br>189pEHTVTTTTKGEN*FTETDIK207	✓<br>✓	×<br>×
T17-T19	189 **E**HTVTTTTKGEN*FTETDIKMMER212	×	×	189 **Q**HTVTTTTKGEN*FTETDIKIMER212<br>189pEHTVTTTTKGEN*FTETDIKIMER212	✓<br>✓	×<br>×

[a] Not plotted as not very relevant
[b] Unfavourable chromatographic properties (too hydrophilic) do not allow this peptide to be reliably detected
[c] Signal from bovine T14 and T13-T14 has been combined.
[d] Usually very low abundance due to frequently missed R-P cleavage
[e] Only plotted if ovine H154 polymorphism is present
