# Supplementary material for: Molecular characterisation of atypical BSE prions by mass spectrometry and changes following transmission to sheep and transgenic mouse models
Source: PLoS One. 2018 Nov 8;13(11):e0206505. doi: 10.1371/journal.pone.0206505 (PMC6224059; doi:10.1371/journal.pone.0206505)
Supplement: S2 Table — The semi-tryptic peptides are the product of N-terminal cleavage by PK of tryptic peptide T7-T9, a sequence which includes two KP missed cleavages. Amino acid residues in bold: bovine/ovine interspecies polymorphisms. pE denotes a pyroglutamyl N-terminal amino acid residue. (PDF) [file pone.0206505.s011.pdf]

S2 Table. Bovine and ovine PrP semi-tryptic peptides used for N-terminal amino acid profiling. The semi-tryptic peptides are the product of N-terminal cleavage by PK of tryptic peptide T7-T9, a sequence which includes two KP missed cleavages. Amino acid residues in bold: bovine/ovine interspecies polymorphisms. pE denotes a pyroglutamyl N-terminal amino acid residue.

N-terminus	Bovine	In Assay	Plotted	Ovine	In Assay	Plotted
G77	GQPHGGGWGQPHGGGGWGQGG**T**H**G**QWNKPSKPK	✓	✓	GQPHGGGWGQPHGGGGWGQGG**S**H**S**QWNKPSKPK	✓	✓
G81	GGGWGQPHGGGGWGQGG**T**H**G**QWNKPSKPK	✓	✓	GGGWGQPHGGGGWGQGG**S**H**S**QWNKPSKPK	✓	✓
G85	GQPHGGGGWGQGG**T**H**G**QWNKPSKPK	✓	✓	GQPHGGGGWGQGG**S**H**S**QWNKPSKPK	✓	✓
Q86<br>pE86	QPHGGGGWGQGG**T**H**G**QWNKPSKPK<br>pEPHGGGGWGQGG**T**H**G**QWNKPSKPK	✓<br>✓	✓<br>✓	QPHGGGGWGQGG**S**H**S**QWNKPSKPK<br>pEPHGGGGWGQGG**S**H**S**QWNKPSKPK	✓<br>✓	✓<br>✓
P87	PHGGGGWGQGG**T**H**G**QWNKPSKPK	×	×	PHGGGGWGQGG**S**H**S**QWNKPSKPK	✓	×
H88	HGGGGWGQGG**T**H**G**QWNKPSKPK	×	×	HGGGGWGQGG**S**H**S**QWNKPSKPK	✓	×
G89	GGGGWGQGG**T**H**G**QWNKPSKPK	✓	✓	GGGGWGQGG**S**H**S**QWNKPSKPK	✓	✓
G90	GGGWGQGG**T**H**G**QWNKPSKPK	✓	✓	GGGWGQGG**S**H**S**QWNKPSKPK	✓	✓
G91	GGWGQGG**T**H**G**QWNKPSKPK	✓	✓	GGWGQGG**S**H**S**QWNKPSKPK	✓	✓
G92	GWGQGG**T**H**G**QWNKPSKPK	✓	✓	GWGQGG**S**H**S**QWNKPSKPK	✓	✓
W93	WGQGG**T**H**G**QWNKPSKPK	✓	✓	WGQGG**S**H**S**QWNKPSKPK	✓	✓
G94	GQGG**T**H**G**QWNKPSKPK	✓	✓	GQGG**S**H**S**QWNKPSKPK	✓	✓
Q95<br>pE95	QGG**T**H**G**QWNKPSKPK<br>pQGG**T**H**G**QWNKPSKPK	✓<br>✓	✓<br>✓	QGG**S**H**S**QWNKPSKPK<br>pEGG**S**H**S**QWNKPSKPK	✓<br>✓	✓<br>✓
G96	GG**T**H**G**QWNKPSKPK	✓	✓	GG**S**H**S**QWNKPSKPK	✓	✓
G97	G**T**H**G**QWNKPSKPK	✓	✓	G**S**H**S**QWNKPSKPK	✓	✓
T98/S98	**T**H**G**QWNKPSKPK	✓	✓	**S**H**S**QWNKPSKPK	✓	✓
H99	H**G**QWNKPSKPK	✓	✓	H**S**QWNKPSKPK	✓	✓
G100/S100	**G**QWNKPSKPK	✓	✓	**S**QWNKPSKPK	✓	✓
Q101<br>pE101	QWNKPSKPK<br>pEWNKPSKPK	✓<br>✓	✓<br>✓	QWNKPSKPK<br>pEWNKPSKPK	✓<br>✓	✓<br>✓
W102	WNKPSKPK	✓	✓	WNKPSKPK	✓	✓
N103	NKPSKPK[a]	✓	×	NKPSKPK	✓	×

[a] Unfavourable chromatographic properties (too hydrophilic) do not allow this peptide to be reliably detected
