# Supplementary material for: Molecular characterisation of atypical BSE prions by mass spectrometry and changes following transmission to sheep and transgenic mouse models
Source: PLoS One. 2018 Nov 8;13(11):e0206505. doi: 10.1371/journal.pone.0206505 (PMC6224059; doi:10.1371/journal.pone.0206505)
Supplement: S3 Table — The LoD was defined as the concentration above which peptides could be detected with a signal-to-noise ratio larger than 3. The LlOQ was defined as the concentration above which the mean concentration determined for three QC standards was within 20% of the expected concentration. ND = not determined (returned values were outside required range). The equivalent values for ovine peptides have been published previously [35]. (PDF) [file pone.0206505.s012.pdf]

S3 Table. Average (av) molecular mass, precursor and product ions used in assay, and limits of detection (LoDs) and lower limits of quantification (LoQs) for bovine PrP peptides included in the assay. The LoD was defined as the concentration above which peptides could be detected with a signal-to-noise ratio larger than 3. The LlOQ was defined as the concentration above which the mean concentration determined for three QC standards was within 20% of the expected concentration. ND= not determined (returned values were outside required range). The equivalent values for ovine peptides have been published previously [35].

Peptide	Molecular Mass (av)	Precursor m/z	Product m/z	LoD (fmol/µl)	LoQ (fmol/µl)
G77-K109	3317.6	554.0<br>554.0<br>475.0	627.5<br>623.9<br>523.1	2	ND
G81-K109	2898.1	580.7<br>580.7<br>484.1	697.0<br>636.2<br>508.2	2	ND
G85-K109	2540.7	509.2<br>509.2<br>424.6	589.8<br>505.7<br>508.2	2	10
Q86-K109	2483.7	497.0<br>415.0<br>415.0	493.9<br>508.1<br>412.0	0.5	ND
pE86-K109	2100.8	494.2<br>494.2<br>494.2	469.7<br>508.0<br>366.8	5	10
G89-K109	2121.3	531.4<br>531.4<br>425.3	632.1<br>569.9<br>508.2	1	1
G90-K109	2064.3	517.1<br>413.9<br>413.9	512.5<br>545.2<br>508.2	5	5
G91-K109	2007.2	502.8<br>502.8<br>502.8	569.8<br>474.2<br>470.0	1	1
G92-K109	1950.1	488.6<br>488.6<br>488.6	761.8<br>569.9<br>564.0	0.2	1
W93-K109	1893.1	474.3<br>474.3<br>474.3	671.7<br>569.9<br>470.0	0.5	1
G94-K109	1706.9	427.8<br>427.8<br>342.5	508.2<br>423.4<br>339.0	0.5	1
Q95-K109	1649.8	413.0<br>413.0	408.9<br>492.9	0.2	ND
pE95-K109	1712.9	409.1<br>409.1<br>409.1	649.1<br>508.1<br>489.1	2	2
G96-K109	1521.7	381.5<br>381.5<br>381.5	489.2<br>470.0<br>377.0	0.2	1
G97-K109	1464.6	367.2<br>294.0	362.8<br>352.9	0.5	1

		294.0	290.2		
T98-K109	1407.6	353.0<br>353.0<br>282.6	556.4<br>348.6<br>279.0	0.5	1
H99-K109	1306.5	436.5<br>327.7<br>327.7	623.2<br>556.4<br>323.4	10	10
G100-K109	1169.3	390.8<br>390.8<br>293.4	492.9<br>385.0<br>289.0	0.2	1
Q101-K109	1112.3	371.5<br>371.5<br>278.9	556.6<br>365.9<br>365.7	0.2	1
pE101-K109	1095.2	365.9<br>365.9<br>365.9	556.3<br>459.3<br>360.3	0.2	1
W102-K109	984.1	328.9<br>328.9	556.2<br>323.1	0.1	1
H114-R139<br>(T11)	2331.7	777.7<br>777.7	862.5<br>608.2	0.1	5
P140-R151<br>(T12)	1448.6	483.8<br>483.8<br>483.8	620.0<br>563.4<br>477.7	1	1
E155-R159<br>(T14)	685.8	343.8<br>343.8<br>343.8<br>229.6<br>229.6	568.3<br>443.3<br>334.8<br>223.6<br>217.9	2	5
Y160-R167<br>(T15)	1102.2	551.8<br>551.8	842.4<br>470.2	0.5	1
P168-K188<br>(T16)	2539.65	635.8<br>635.8	804.5<br>440.1	5	5
G198-K207<br>(T18)	1153.2	577.3<br>577.3	967.4<br>706.3	1	1
V212-R223<br>(T20)	1602.7	535.0<br>535.0	752.0<br>620.0	0.1	1
E224-R231<br>(T21)	1044.1	522.9<br>522.9	629.3<br>466.1	0.5	1
Y152-R159<br>(T13-14)	1168.3	390.5<br>390.5<br>293.2	503.4<br>384.7<br>336.0	2	2
Y160-K188<br>(T15-16)	3624.0	725.8<br>725.8<br>725.8	762.7<br>722.4<br>698.7	1	1
