# Supplementary material for: Molecular characterisation of atypical BSE prions by mass spectrometry and changes following transmission to sheep and transgenic mouse models
Source: PLoS One. 2018 Nov 8;13(11):e0206505. doi: 10.1371/journal.pone.0206505 (PMC6224059; doi:10.1371/journal.pone.0206505)
Supplement: S4 Table — Bold: significant difference (P< 0.05), Bold italic (P<0.01). Outlined areas as referred to in text. Table A: P-values for differences between BSE types: T16/T18 Table B: P-values for differences between BSE types: T16/T20 Table C: P-values for differences between BSE types: T18/T20 (PDF) [file pone.0206505.s013.pdf]

**S4 Table.** P-values for two-tailed t-test for significant differences between glycoforms ratios. **Bold**: significant difference (P< 0.05), ***Bold italic*** (P<0.01). Outlined areas as referred to in text.

Table A: P-values for differences between BSE types: T16/T18

| | Bov C | Bov H | Bov L | Tg110 C | Tg1896 C | Tg110 H | Tg1896 H | Tg110 L | Tg1896 L | Ov L |
|---|---|---|---|---|---|---|---|---|---|---|
| Bov C | x | **0.0744** | **0.0175** | ***< 0.0001*** | ***0.0023*** | 0.1498 | 0.7197 | 0.8595 | 0.2756 | ***0.0085*** |
| Bov H | | x | **0.0440** | ***< 0.0001*** | ***0.0018*** | 0.9344 | 0.1591 | 0.1506 | 0.5995 | 0.5118 |
| Bov L | | | x | 0.1546 | 0.7727 | 0.1282 | 0.4433 | 0.2458 | 0.1484 | **0.0209** |
| Tg110 C | | | | x | **0.0141** | ***0.0005*** | **0.0171** | ***0.0015*** | ***0.0005*** | ***< 0.0001*** |
| Tg1896 C | | | | | x | ***0.0067*** | 0.1502 | 0.0538 | ***0.0072*** | ***< 0.0001*** |
| Tg110 H | | | | | | x | 0.0810 | 0.1677 | 0.4447 | 0.2734 |
| Tg1896 H | | | | | | | x | 0.8450 | **0.0395** | 0.0019 |
| Tg110 L | | | | | | | | x | 0.2356 | 0.0120 |
| Tg1896 L | | | | | | | | | x | 0.0183 |
| Ov L | | | | | | | | | | x |

Table B: P-values for differences between BSE types: T16/T20

| | Bov C | Bov H | Bov L | Tg110 C | Tg1896 C | Tg110 H | Tg1896 H | Tg110 L | Tg1896 L | Ov L |
|---|---|---|---|---|---|---|---|---|---|---|
| Bov C | x | 0.0823 | ***0.0020*** | ***< 0.0001*** | **0.0107** | 0.5276 | 0.0790 | ***0.0020*** | 0.1128 | **0.0429** |
| Bov H | | x | 0.1644 | 0.5852 | 0.6718 | 0.2953 | 0.7528 | 0.9664 | 0.5437 | 0.4911 |
| Bov L | | | x | 0.3749 | 0.1809 | 0.0800 | 0.3447 | 0.2470 | 0.1557 | 0.0521 |
| Tg110 C | | | | x | 0.0036 | ***0.0001*** | **0.0279** | 0.2076 | ***0.0011*** | **0.0073** |
| Tg1896 C | | | | | x | ***0.0029*** | 0.8009 | 0.3579 | 0.0712 | 0.8139 |
| Tg110 H | | | | | | x | **0.0216** | **0.0330** | **0.0110** | 0.1059 |
| Tg1896 H | | | | | | | x | 0.5016 | 0.1141 | 0.9361 |
| Tg110 L | | | | | | | | x | 0.1817 | 0.2610 |
| Tg1896 L | | | | | | | | | x | 0.6886 |
| Ov L | | | | | | | | | | x |

Table C: P-values for differences between BSE types: T18/T20

| | Bov C | Bov H | Bov L | Tg110 C | Tg1896 C | Tg110 H | Tg1896 H | Tg110 L | Tg1896 L | Ov L |
|---|---|---|---|---|---|---|---|---|---|---|
| Bov C | x | ***0.0005*** | **0.0203** | 0.2237 | 0.3676 | ***0.0008*** | ***0.0008*** | ***< 0.0001*** | ***< 0.0001*** | ***< 0.0001*** |
| Bov H | | x | 0.4305 | 0.0710 | 0.0733 | 0.0818 | 0.3098 | 0.3378 | 0.2721 | 0.8863 |
| Bov L | | | x | 0.2301 | 0.2366 | 0.3078 | 0.5902 | 0.8195 | 0.7015 | 0.2392 |
| Tg110 C | | | | x | 0.7261 | ***0.0010*** | ***0.0026*** | ***< 0.0001*** | ***< 0.0001*** | ***0.0003*** |
| Tg1896 C | | | | | x | ***0.0036*** | ***0.0099*** | ***< 0.0001*** | ***< 0.0001*** | ***0.0003*** |
| Tg110 H | | | | | | x | 0.2353 | ***< 0.0001*** | ***< 0.0001*** | ***0.0003*** |
| Tg1896 H | | | | | | | x | ***0.0067*** | **0.0103** | **0.0191** |
| Tg110 L | | | | | | | | x | 0.0702 | **0.0185** |
| Tg1896 L | | | | | | | | | x | ***0.0093*** |
| Ov L | | | | | | | | | | x |
